# Evaluating finger-prick blood collection for remote quantification of neurofilament light in neurological diseases

**DOI:** 10.1007/s00415-025-13232-8

**Published:** 2025-07-10

**Authors:** Annabelle Coleman, Alexiane Touzé, Mena Farag, Marta Pengo, Michael J. Murphy, Yara Hassan, Olivia Thackeray, Kate Fayer, Sophie Field, Mitsuko Nakajima, Elizabeth L. Broom, Nicola Z. Hobbs, Brook Huxford, Natalie Donkor, Ellen Camboe, Kamalesh C. Dey, Alexandra Zirra, Aisha Ahmed, Ana Rita Gameiro Costa, Harriet Sorrell, Luca Zampedri, Vittoria Lombardi, Charles Wade, Sean Mangion, Batoul Fneich, Amanda Heslegrave, Henrik Zetterberg, Rachael Scahill, Alastair Noyce, Andrea Malaspina, Jeremy Chataway, Sarah J. Tabrizi, Lauren M. Byrne

**Affiliations:** 1 UCL Huntington’s Disease Centre, UCL Queen Square Institute of Neurology, Queen Square, London, WC1N 3BG UK; 2 Department of Molecular and Translational Medicine, University of Brescia, 25123 Brescia, Italy; 3 San Paolo University Hospital, ASST Santi Paolo e Carlo, Milan, Italy; 4 Centre for Preventive Neurology, Wolfson Institute of Population Health, Queen Mary University of London, London, EC1M 6BQ UK; 5 Royal London Hospital, Barts Health NHS Trust, London, E1 1FR UK; 6 Queen Square MND Centre, UCL Queen Square Institute of Neurology, Queen Square, London, WC1N 3BG UK; 7 The United to End MND (U2EM) / UK MND Research Institute Consortium, London, UK; 8 National Hospital for Neurology and Neurosurgery, University College London Hospitals, Queen Square, London, WC1N 3BG UK; 9 University College London Hospital, 250 Euston Road, London, NW1 2A UK; 10 Queen Square Multiple Sclerosis Centre, Department of Neuroinflammation, UCL Queen Square Institute of Neurology, Faculty of Brain Sciences, University College London, London, WC1B 5EH UK; 11 UK Dementia Research Institute and Department of Neurodegenerative Diseases, UCL Queen Square Institute of Neurology, Queen Square, London, WC1N 3BG UK; 12 Department of Psychiatry and Neurochemistry, Institute of Neuroscience and Physiology, The Sahlgrenska Academy at the University of Gothenburg, Mölndal, Sweden; 13 Clinical Neurochemistry Laboratory, Sahlgrenska University Hospital, Mölndal, Sweden; 14 Hong Kong Center for Neurodegenerative Diseases, Clear Water Bay, Hong Kong, China; 15 Wisconsin Alzheimer’s Disease Research Center, University of Wisconsin School of Medicine and Public Health, University of Wisconsin-Madison, Madison, WI 53792 USA; 16 National Institute for Health Research, University College London Hospitals, Biomedical Research Centre, London, W1T 7DN UK

**Keywords:** Neurodegenerative disorders, Biomarkers, Neurofilament light, Blood, Remote sampling, Finger-prick

## Abstract

Promising blood-based biomarkers of neuropathology have emerged with potential for therapeutic development and disease monitoring. However, these tools will require specialist tertiary services for integration into clinical management. Remote sampling for biomarker assessment could reduce burden of in-person clinical visits for such tests as well as increasing the sampling frequency and patient geographical outreach. Here, we evaluated a finger-prick blood collection approach for remote quantification of neurofilament light (NfL), a candidate blood-based biomarker evident in various neurological disorders, and other exploratory markers of neuronal injury and neuroinflammation (GFAP, tau). Matched samples from venepuncture and finger-prick were collected and processed into plasma and/or serum to directly compare analyte levels from a multi-disease discovery cohort (*n = *54 healthy controls; *n = *57 Huntington’s disease (HD); *n = *34 multiple sclerosis; *n = *7 amyotrophic lateral sclerosis; *n = *11 Parkinson’s disease), and a HD confirmatory cohort (*n = *57 healthy controls; *n = *64 HD). Two delayed processing conditions were compared, three- and seven-day delay, simulating ambient shipment. Capillary NfL and GFAP concentrations were equivalent to those in venous serum and plasma in the multi-disease discovery cohort and HD confirmatory cohort. Only NfL remained stable after a seven-day processing delay in both venous and capillary serum samples. Using NfL concentrations from capillary blood, we replicated previously published disease group differences measured in venous blood. This data supports our finger-prick approach for remote collection and quantification of NfL. With the widespread applications for NfL across the spectrum of neurological disorders, this has the potential to transform disease monitoring, prognosis, and therapeutic development within clinical practice and research.

**Supplementary Information:**

The online version contains supplementary material available at 10.1007/s00415-025-13232-8.

## Background

Blood-based biomarkers with pathobiological relevance to the central nervous system have emerged over recent years with strong potential for clinical applications [1–3]. However, neurological disorders tend to be managed within tertiary care settings, requiring patients to travel to specialised medical centres for multidisciplinary care. Monitoring of neuropathological blood tests would likely require specialist neurological review. While specialist services offer more bespoke care for these complex conditions, frequent clinic visits for blood tests or MRI scans could intensify the burden on patients and their caregivers. The ability for patients to collect their own blood at home for regular monitoring of such biomarkers would therefore be highly desirable.

As we move closer to prevention trials for neurodegenerative diseases [4] the implications of treating presymptomatic individuals with neuropathological changes need to be considered for clinical trial design. The targeted demographics for such trials will encompass younger individuals who maintain full functional abilities, despite their risk of developing a neurodegenerative disease. These individuals carry their own responsibilities, including childcare and early careers, which pose challenges to attending the many in-person visits required for clinical trial participation. Remote sampling for biomarker assessment offers several key benefits: (1) The convenience of self-sampling at home makes repeated sampling easier on participants, which in turn could reduce dropout in long-term longitudinal studies or trials; (2) Remote sampling could overcome geographical barriers for individuals in areas where a specialist centre is inaccessible; (3) Remote assessments could effectively address challenges associated with recruitment of ultrarare populations. For example, remote testing for sexually transmitted infections almost doubled uptake among ‘never-testers’ [5]. Sexual Health London (SHL) and several commercial companies actively provide remote self-sampling services to collect capillary blood via a finger-prick into 600 µL microtainer tubes equipped with additives tailored to the post-processing requirements of the desired analytes. Examples include lithium heparin (LiHep) tubes for plasma and serum separator tubes (SST) for serum.

A candidate blood-based biomarker implicated in many neurological disorders is neurofilament light protein (NfL), a marker of ongoing neuronal damage, which can be measured in both cerebrospinal fluid (CSF) and blood [6]. Natural history studies have demonstrated elevated NfL levels in comparison to age-matched healthy controls in Huntington’s disease (HD) [7, 8], multiple sclerosis (MS) [9], amyotrophic lateral sclerosis (ALS) [10], frontotemporal dementia [11], and Alzheimer’s disease [12], including those in the very early stages of disease [13, 14]. Plasma NfL levels have also demonstrated the ability to differentiate idiopathic Parkinson’s disease (PD) from atypical Parkinsonian syndromes [15]. NfL also shows potential as a surrogate endpoint with reductions following efficacious treatment demonstrated in MS [16] and spinal muscular atrophy [17]. Recently, an anti-sense oligonucleotide therapeutic, Tofersen, targeting SOD1 received FDA accelerated approval based on the ability of the drug to lower blood NfL levels in patients with SOD1 mutation-mediated ALS in combination with signs of clinical benefit [18].

There have been previous efforts to use small volumes of venous blood to measure NfL to aid remote monitoring, including dried plasma spots (DPS) using Noviplex Plasma Prep cards [19, 20]. Several studies have demonstrated that NfL is highly stable in venous blood plasma and can withstand freeze-thawing and delayed sample processing, even up to seven days [21, 22]. However, no studies have empirically tested whether NfL concentrations measured in capillary plasma or serum align with those observed in the established gold standard of venous plasma or serum.

In this study, we adapted a previously used finger-prick blood collection approach and applied it to four neurological conditions (MS, HD, ALS, PD) to assess the validity of quantifying NfL and other exploratory markers of neuronal injury and inflammation via remote collection. In a multi-disease discovery cohort and an HD confirmatory cohort, we collected matched samples from venepuncture and finger-prick and processed them into plasma and/or serum to directly compare analyte concentrations between the collection types. In the multi-disease discovery cohort, we compared two delayed processing conditions, three- and seven-day delay, and simulated ambient shipment. We report analyte concentrations from capillary plasma and serum and evidence to support NfL’s application for at-home testing.

## Methods

### Study design

This cross-sectional study was designed to use a finger-prick blood collection approach and technically validate it for remote quantification of NfL in a multi-disease discovery cohort of four neurological conditions (MS, HD, ALS, and PD) and in an HD confirmatory cohort. Participants with these neurological conditions were included to (1) technically validate the collection approach across a broad range of healthy and diseased NfL concentrations, and (2) assess whether this collection approach is feasible for individuals with these neurological conditions.

**Fig. 1:**
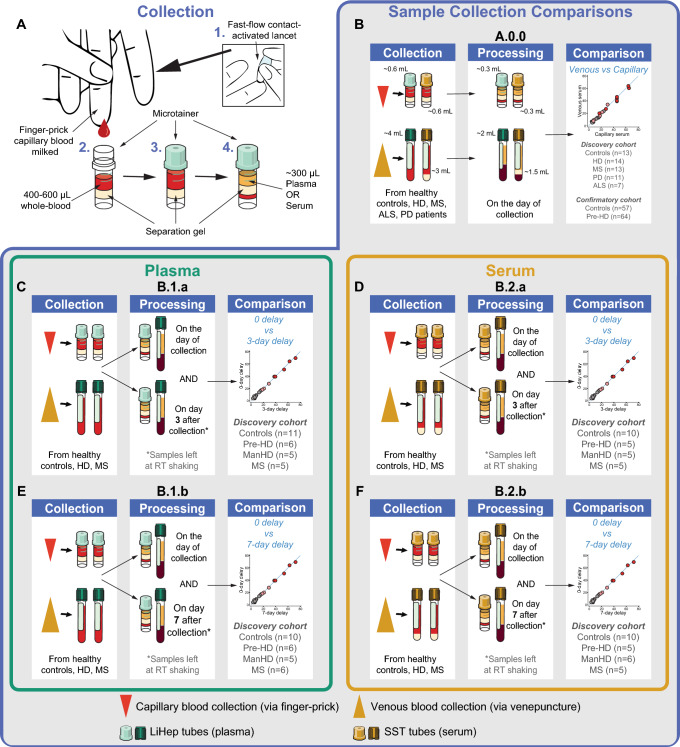
Study design and experimental objectives. **A** Schematic showing capillary sample collection method: 1. Finger-prick performed using a fast-flow lancet, 2. Blood milked from finger, 3. Up to 600 µL whole blood collected into two LiHep and/or SST microtainers, 4. ~ 300 µL capillary plasma/serum generated after processing per microtainer. **B** Experimental objective A.0.0: matched capillary (microtainer) and venous (vacutainer) samples were collected in serum and plasma tubes and processed on the day of collection to compare analytes between collection methods (finger-prick/venepuncture) and sample types (plasma/serum) in both the multi-disease discovery cohort and HD confirmatory cohort. **C** Experimental objective B.1.a: assessing the impact of 3-day processing delay in plasma. **D** Experimental objective B.2.a: assessing the impact of 3-day processing delay in serum. **E** Experimental objective B.1.b: assessing the impact of 7-day processing delay in plasma. **F** Experimental objective B.2.b: assessing the impact of 7-day processing delay in serum. For delayed processing experiments, **C**–**F** two tubes were collected from both finger-prick and venepuncture for either plasma or serum and one tube of each collection type was processed on day zero and the other on the respective processing delay day after shaking at room temperature. Green tubes indicate plasma and yellow tubes indicate serum. *ALS* amyotrophic lateral sclerosis; *HD* Huntington’s disease; *MS* multiple sclerosis; *PD* Parkinson’s disease; *Pre-HD* premanifest HD; *RT* room temperature

Five experiments (Fig. 1) were designed and applied to the multi-disease discovery cohort: Experiment A.0.0 compared the impact of collection method (finger-prick versus venepuncture) and sample type (plasma versus serum) on analyte concentrations. The impact of delayed processing on analyte concentrations was evaluated by comparing concentrations before and after a three- or seven-day processing delay for both collection methods and sample types (experiment B.1.a: 3-day plasma; B.2.a: 3-day serum; B.1.b: 7-day plasma; B.2.b: 7-day serum). Experiment A.0.0 was replicated in the HD confirmatory cohort. For all experiments, total protein and haemoglobin were quantified to assess sample quality and the impact of delayed processing on protein degradation and haemolysis, respectively. Glial fibrillary acidic protein (GFAP) and total Tau (tTau) were quantified as exploratory biomarkers for remote quantification.

### Participant recruitment

For the multi-disease discovery cohort, healthy control and HD participants were recruited from the National Hospital for Neurology and Neurosurgery (NHNN) HD multidisciplinary clinic, part of University College London (UCL) Hospitals NHS Foundation Trust. Healthy controls were family members without disease risk or members of the broader HD community. HD mutation carriers required a positive genetic test for HD (CAG ≥ 37) and included those before clinical motor onset (pre-HD) with no motor signs or symptoms, and those with clinical motor onset (manifest-HD) required a Unified Huntington’s Disease Rating Scale (UHDRS) Diagnostic Confidence Level (DCL) of 4 [23]. ALS-slow and ALS-fast participants were recruited from the Lighthouse II study, ALS biomarkers study, and NHNN ALS clinic. ALS phenotypic stratification was based on disease progression rate, calculated as the slope of functional decline in ALS Functional Rating Scale–Revised (ALSFRS-R) [24] scores from symptom onset to sample collection. The rate of decline (ΔFRS) was defined as the change in score divided by the number of months between these time points. Fast progressors (ALS-fast) had ΔFRS ≥ 0.8, while slow progressors (ALS-slow) had ΔFRS ≤ 0.5. Relapsing–remitting, primary progressive, and secondary progressive MS participants were recruited from the UCL Institute of Neurology Clinical Trials (MS-STAT2 and Octopus) and NHS outpatients. MS subgroups were determined according to the 2017 McDonald criteria [25]. PD participants with a clinical diagnosis of PD, made by experienced movement disorders consultants [26–28], were recruited from the East London Parkinson’s Disease project, Royal London Hospital and Barts Health NHS Trust. Exclusion criteria for PD included drug-induced parkinsonism, vascular parkinsonism, atypical parkinsonism, and other significant neurological or psychiatric comorbidity. All participants were aged 18–84 years, able to tolerate blood collection, and without major psychiatric disorder or history of significant head injury.

For the HD confirmatory cohort, healthy controls and pre-HD individuals were recruited from the second timepoint of a unique presymptomatic HD cohort called the HD Young Adult Study (HD-YAS 2.0) [14, 29]. Pre-HD participants required a positive genetic test for HD (CAG ≥ 40) but had no clinical signs or symptoms. Exclusions included MRI contraindications, significant comorbidities, untested at-risk status, and reduced penetrance *HTT* CAG repeat length (36–39). Controls were gene-negative individuals with a family history of HD, family members without disease risk, or members of the broader HD community. The full study protocol can be found at Scahill et al., (2020 & 2025) [14, 29].

### Blood collection and processing

Venous blood was collected via venepuncture using a butterfly needle. We used a finger-prick approach using a fast-flow lancet (Product code: 366594) to collect capillary blood (Fig. 1A). Capillary whole blood was gently milked and collected in serum and plasma microtainer tubes (400–600 µL per tube; BD SST serum gold top [Product code: 365968] or BD PST LiHep plasma green top [Product code: 12957646]).

Blood from both vacutainers and microtainers was processed using the manufacturer's recommended settings on-site to isolate serum or plasma, either on the day of, three, or seven days after sample collection. Vacutainer tubes were centrifuged at 1300*g* for 10 min at room temperature, and the supernatant was isolated and aliquoted into 500 µL aliquots. Microtainers were centrifuged at 15,000*g* for 2 min at room temperature and the supernatant was isolated, aliquoted into 100 µL aliquots. Processed samples were then stored at − 70 °C until analyte quantification.

Delayed processing experiments to assess the impact of delayed processing on NfL in both plasma and serum included collecting two tubes of either plasma or serum for both capillary and venous blood collection. One set of capillary and venous tubes was processed immediately on the collection day, while the other set was left on a shaker (24 rpm) for either three or seven days before processing to simulate delays from ambient shipment of at-home collected samples.

### Analyte quantification

NfL, GFAP, and tTau concentrations were quantified using a commercially available Neurology 4-Plex-B (N4PB) kit on the Simoa HD-X analyser platform following the manufacturer’s instructions (Quanterix, Billerica, MA). Controls and samples were run in duplicates with machine dilution of 1:4. Haemoglobin was measured in duplicates using a commercial ELISA kit (Bethyl Laboratories, cat#E88-134) according to the manufacturer’s specifications. Samples processed on the day of collection and three days later were diluted 1:3000 and samples that were processed seven days after collection were diluted 1:27,000 to keep samples in the linear range of the assay. Total protein was also measured using a commercial ELISA kit (Pierce™ BCA Protein Assay Kits, cat#23227) according to the manufacturer’s instructions, with samples diluted 1:100. Total protein and haemoglobin were measured for each sample from the same aliquot as the N4PB analysis.

For all analytes, each sample within an objective was run using the same batch of reagents and samples from the same individual were run on the same plate. The inter-assay Coefficients of Variance (CV) and average intra-assay CVs for each analyte are represented in Supplementary Table 1. All analyte concentrations fell within the linear range of each assay. Quantification of analytes was performed blinded to disease status.

### Statistical analysis

Analyses were performed using Stata/MP 18.0. The significance level was defined as *p < *0.05. Sample sizes were derived using a one-sample correlation test (alpha = 0.01, power = 0.9) based on observed correlations of NfL between venous blood and CSF (*r = *0.7–0.9) [8]. *N = *23 had sufficient power to detect a correlation of *r = *0.7 or higher. Therefore, for each objective, at least 23 participants were recruited (total N sum of all disease groups).

Normality was assessed visually and using Shapiro–Wilk tests for each comparison grouping in all experiments. For the multi-disease discovery cohort, NfL and tTau were non-normally distributed for each sample type and collection method, so natural log-transformed values were used for all experiments. GFAP had non-normal distributions for groups in experiments A.0.0, B.1.a, and B.1.b which were resolved by natural log transformation. For the HD confirmatory cohort, NfL, GFAP, and tTau were non-normally distributed for each sample type and collection method, so natural log-transformed values were used.

For group demographics by experiment, age was analysed using a two-sample *t*-test for comparisons between two groups or a Kruskal–Wallis test when comparing more than two groups. Sex group differences were assessed using Pearson’s *χ*^2^ test.

Outliers were assessed using box plots and spike plots of the raw data; one participant was identified with NfL concentrations more than eight times the second highest value (653 pg/mL and 77 pg/mL, respectively) and a GFAP value more than 26 times the second highest value (7789 pg/mL and 290 pg/mL, respectively). Therefore, this participant was excluded from all further analyses.

Relationships between analyte concentrations and sample types, collection method, and/or delayed processing regimens on analyte concentration for each objective were assessed using linear regression models (*R*^2^) and Pearson’s correlations (*r*). All *p* values were adjusted for multiple comparisons by defining Bonferroni-corrected thresholds for each experiment. Two-way mixed-effects model intraclass correlation coefficients (ICC) were used to measure the agreement of analyte measurements in each objective, with values ranging from zero to one. Bland–Altman plots were also presented to visualise the agreement between measurements across analyte concentrations for each objective. Where there was a visible increase or decrease between analyte values across sample types/collection methods, we performed post-hoc two-tailed paired t-tests to assess whether the difference was significant. For log-transformed values, we report the back-transformed mean difference (MD) and 95% confidence intervals (CI).

To explore group analyte differences between clinical disease groups and controls in the multi-disease discovery cohort, we used all baseline samples across all objectives for each sample type and collection method: day zero concentrations for venous and capillary plasma and serum. Only NfL and GFAP were assessed for intergroup differences due to their high correlations between sample and collection types. Natural log transformations of NfL and GFAP were used due to non-normally distributed data across sample types and groups. Potentially confounding demographic variables (age and sex) were examined in preliminary analyses using multiple linear regression with control data; age was identified as a confounding variable and was included as a covariate in subsequent analyses. Multiple linear regression with post-estimation Wald tests was used to assess intergroup analyte concentrations for each sample type applying Bonferroni correction for multiple comparisons.

To explore disease group differences in NfL concentrations between healthy controls and pre-HD individuals in the HD confirmatory cohort, multiple linear regression adjusting for age was used with Bonferroni correction for multiple comparisons. We conducted further statistical analyses on capillary serum and plasma NfL concentrations in the HD confirmatory cohort, aiming to replicate previous findings of venous blood NfL’s performance as a biomarker in this cohort using capillary blood NfL, including demonstrating whether NfL increases in a CAG-dependent manner. We employed multiple linear regression to model NfL concentrations with age for a range of CAG repeat lengths (40–46). We began by integrating NfL data from the HD-CSF study [8, 30] to capture the entire HD spectrum of NfL concentrations. To identify the best-fitting model, we iteratively assessed models starting with all terms in the full expansion of a polynomial function of age and CAG and then applied backward variable elimination. Subsequently, we fit the model specifically to the HD confirmatory cohort data, accounting for the main effects of age, CAG, the quadratic effect of age, and the interaction between age and CAG.

## Results

### Adapting a finger-prick collection approach for remote NfL quantification

By optimising a finger-prick collection approach, previously used in other fields, we were consistently able to collect two microtainer tubes of capillary blood (600 µL each) from each individual finger-prick session. For this reason, each experimental condition needed to be assessed in independent collections. Initially, we set out to compare our optimised finger-prick approach with previously reported alternative remote collection methods for NfL quantification, including dried blood spots (DBS) and dried plasma spots (DPS) [19, 20, 31]. However, previous studies demonstrated a notable reduction in venous NfL concentrations when quantified from DPS/DBS in comparison to the established gold standard of venous plasma [19, 20, 31]. Additionally, these methods have not been utilised for quantifying NfL levels in capillary blood. Therefore, we conducted a pilot collection to compare NfL concentrations from venous and capillary plasma (*n = *4 healthy controls, *n = *4 pre-HD participants, and *n = *4 manifest-HD participants). The results indicated plasma collected from finger-prick produced NfL concentrations more similar to that from the gold standard (venepuncture) than previously published alternative methods, such as DBS and DPS [19, 20, 31] (Supplementary Fig. 1a–i). Hence, we went on to simply compare our finger-prick plasma and serum collection approach to that from the venous blood gold standard. The group demographics for each experimental objective are summarised in Supplementary Table 2. We quantified total protein and haemoglobin for quality control assessment of each collection method and sample type. GFAP and tTau were quantified as exploratory markers of interest. Summary statistics of all analytes for each experiment are presented in the supplementary material (Supplementary Tables 3–7).

### NfL and GFAP concentrations are highly correlated in venous and capillary serum and plasma in the multi-disease discovery cohort

Total protein and haemoglobin concentrations in capillary and venous blood from the multi-disease discovery cohort were highly variable across collection methods and sample types (Supplementary Fig. 2a–j). These assays had similarly low technical variability within runs (intra-assay CV) as those quantified by the ultrasensitive Simoa method (Supplementary Table 1). Total protein and haemoglobin showed high inter-assay CVs however, because each participant’s samples were run within the same plate, this should not have affected the within-subject comparisons. There were significantly higher concentrations of total protein in venous blood compared to capillary (Plasma: Mean Difference (MD) = 9 mg/mL, 95% Confidence Interval (CI) 6.5–11.5, *p < *0.0001, Supplementary Fig. 3a; Serum: MD = 10.7 mg/mL, 95% CI 8.0–13.4, *p < *0.0001, Supplementary Fig. 3b).

**Fig. 2:**
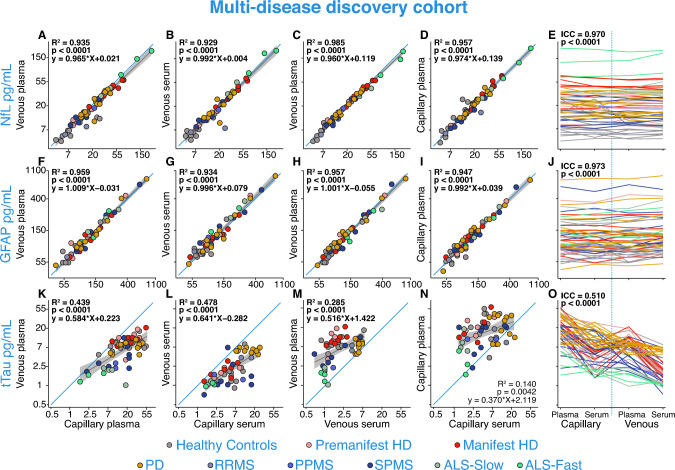
A.0.0: Analyte concentrations in venous versus capillary and plasma versus serum. Analyte concentrations across different collection methods and sample types from experiment A.0.0 for NfL (**A**–**E**), GFAP (**F**–**J**), and tTau (**K**–**O**) in healthy controls, pre-HD, manifest-HD, PD, RRMS, PPMS, SPMS, ALS-slow, ALS-fast. Blue lines represent the line of equality (*y* = *x*). Grey lines represent the linear regression fit of the data with 95% confidence interval shaded regions. *R*^2^ and *p* values were generated from regression models comparing the two collection types in each panel. The Bonferroni threshold for this experiment was 0.0025 (20 comparisons) and all statistics which reached significance below this are highlighted in bold. NfL, GFAP, and tTau concentrations were natural log-transformed. *ALS* amyotrophic lateral sclerosis; *GFAP* glial fibrillary acidic protein; *Haem* haemoglobin; *HD* Huntington’s disease; *NfL* neurofilament light; *PD* Parkinson’s disease; *PPMS* primary progressive multiple sclerosis; *RRMS* relapsing–remitting multiple sclerosis; *SPMS* secondary progressive multiple sclerosis; *tTau* total tau

Despite this, NfL concentrations were strongly correlated between venous and capillary samples in both plasma (*r = *0.967, *R*^2^ = 0.935, 95% CI 0.944–0.980, *p < *0.0001; Fig. 2A) and serum (*r = *0.963, *R*^2^ = 0.929, 95% CI 0.938–0.979, *p < *0.0001; Fig. 2B), and plasma and serum NfL values were equivalent in both collection types (Venous: *r = *0.993, *R*^2^ = 0.985, 95% CI 0.988–0.996, *p < *0.0001; Fig. 2C; Capillary: *r = *0.979, *R*^2^ = 0.957, 95% CI 0.964–0.988, *p < *0.0001; Fig. 2D). This was similar for GFAP concentrations (Plasma: *r = *0.979, *R*^2^ = 0.959, 95% CI 0.965–0.988, *p < *0.0001; Serum: *r = *0.967, *R*^2^ = 0.934, 95% CI 0.944–0.981, *p < *0.0001; Venous: *r = *0.978, *R*^2^ = 0.985, 95% CI 0.963–0.987, *p < *0.0001; Capillary: *r = *0.973, *R*^2^ = 0.957, 95% CI 0.95–0.984, *p < *0.0001; Fig. 2P–S, respectively). NfL and GFAP values across all four samples and collection types compared in experiment A.0.0 showed strong agreement (NfL: ICC = 0.970, 95% CI 0.989–0.995, *p < *0.0001, Fig. 2O; GFAP: ICC = 0.973, 95% CI 0.989–0.996, *p < *0.0001, Fig. 2T) and variance did not change with concentration (Bland–Altman limit of agreement < 1 standard deviation (SD) of the NfL and GFAP mean differences between different collection types; Supplementary Fig. 4i-p). tTau varied more across sample and collection types (ICC = 0.510, 95% CI 0.692–0.872, *p < *0.0001, Supplementary Fig. 3e–h), and concentrations were higher in capillary blood compared to venous blood and in plasma compared to serum. Using a post-hoc paired t-test, these increases were significant (Plasma: MD = 8.78 pg/mL, 95% CI 6.01–12.8, *p < *0.0001, Supplementary Fig. 3e; Serum: MD = 8.4 pg/mL, 95% CI 5.76–12.2, *p < *0.0001, Supplementary Fig. 3f) and were higher in plasma samples compared to serum samples in both collection types (Capillary: MD = 10.2 pg/mL, 95% CI 5.95–17.6, *p < *0.0001, Supplementary Fig. 3g; Venous: MD = 10.5 pg/mL, 95%CI 6.88–15.9, *p < *0.0001, Supplementary Fig. 3h).

### NfL shows high stability after three-day delayed processing in venous and capillary serum and plasma in the multi-disease discovery cohort

**Fig. 3:**
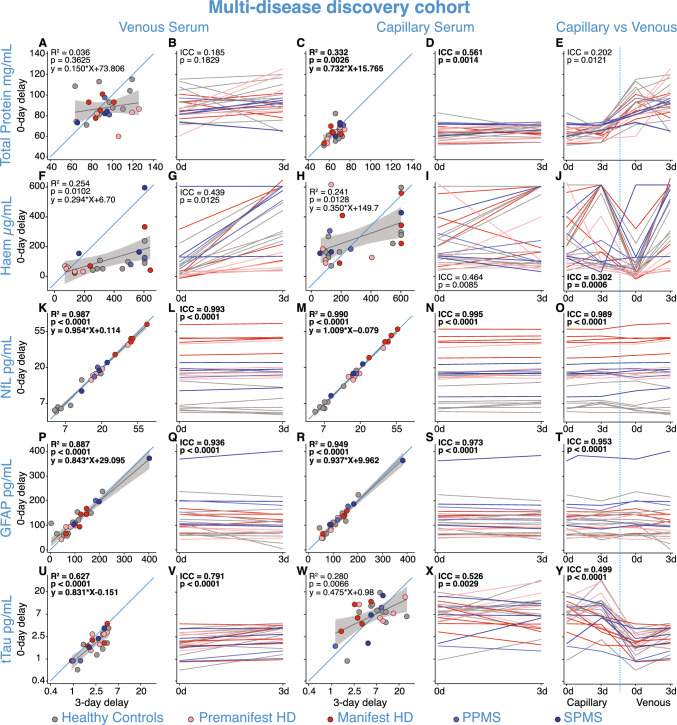
B.2.a: Analyte concentrations in venous/capillary serum with and without 3-day delay in processing. Analyte concentrations in serum after zero-day and three-day delay in processing from experiment B.2.a. in the multi-disease discovery cohort for total protein (**A**–**E**), haemoglobin (**F**–**J**), NfL (**K**–**O**), GFAP (**P**–**T**), and tTau (**U**–**Y**) from venous and capillary serum in healthy controls, pre-HD, manifest-HD, PPMS, SPMS. Blue lines represent the line of equality (*y* = *x*). Grey lines represent the linear regression fit of the data with 95% confidence interval shaded regions. *R*^2^ and *p* values were generated from regression models comparing the impact of delayed processing in each panel. The Bonferroni threshold for this experiment was 0.005 (10 comparisons) and all statistics which reached significance below this are highlighted in bold. NfL and tTau concentrations were natural log-transformed. *GFAP* glial fibrillary acidic protein; *Haem* haemoglobin; *HD* Huntington’s disease; *NfL* neurofilament light; *PPMS* primary progressive multiple sclerosis; *SPMS* secondary progressive multiple sclerosis; *tTau* total tau

After confirming capillary blood NfL was equivalent to venous blood NfL, we tested the impact of a three-day delay in processing on analyte concentrations in plasma (B.1.a) and serum (B.2.a) from healthy controls, HD and MS participants in the multi-disease discovery cohort. Total protein and haemoglobin concentrations in capillary and venous blood remained highly variable across collection methods and sample types in serum (Fig. 3A–J). Only venous haemoglobin showed significant elevation after a three-day delay in processing in serum (Fig. 3F–J; MD = 240.1 ug/mL, 95% CI 163.5–316.6, *p < *0.0001, Supplementary Fig. 5b).

Serum NfL concentrations showed strong positive correlations between samples processed on the day of collection versus three days later for both venous (*r = *0.993, *R*^2^ = 0.987, 95% CI 0.985–0.997, *p < *0.0001, Fig. 3K, L) and capillary (*r = *0.995, *R*^2^ = 0.990, 95% CI 0.989–0.998, *p < *0.0001, Fig. 3M, N) samples, suggesting high stability of NfL after three days of delayed processing, irrespective of collection method. The Bland–Altman analysis confirmed strong agreement across the NfL concentrations measured in both serum and plasma between zero- and three-day samples (Supplementary Fig. 6i–l). Similarly, GFAP concentrations showed strong positive correlations in capillary serum (*r = *0.929, *R*^2^ = 0.949, 95% CI 0.941–0.989, *p < *0.0001, Fig. 3R, S) after three-day delay in processing compared to day zero concentrations. Capillary tTau concentrations with three-day delayed processing were again highly variable in serum samples (Fig. 3X, ICC = 0.526, 95% CI 0.295–0.863, *p = *0.0029). Analyte concentrations in plasma after a three-day delay in processing compared to day zero concentrations were very similar to serum but with more variability in GFAP concentrations (Supplementary Fig. 7 and 8).

### NfL shows high stability after seven-day delayed processing in venous and capillary serum and plasma in the multi-disease discovery cohort

**Fig. 4:**
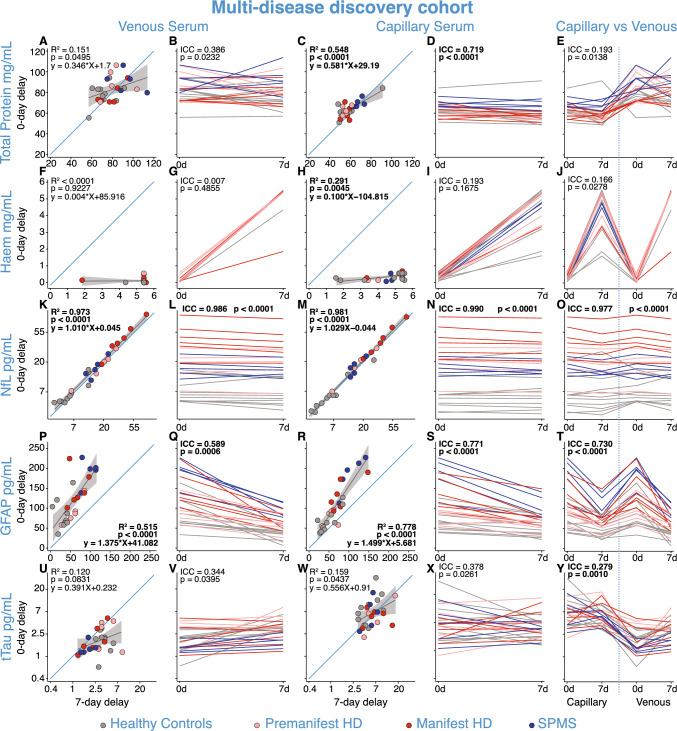
B.2.b: Analyte concentrations in venous/capillary serum with and without 7-day delay in processing. Analyte concentrations in serum after zero-day and seven-day delay in processing from experiment B.2.b for total protein (**A**–**E**), haemoglobin (**F**–**J**), NfL (**K**–**O**), GFAP (**P**–**T**), and tTau (**U**–**Y**) from venous and capillary serum in healthy controls, pre-HD, manifest-HD, and SPMS. Blue lines represent the line of equality (*y* = *x*). Grey lines represent the linear regression fit of the data, with 95% confidence interval shaded regions. *R*^2^ and *p* values were generated from regression models comparing the impact of delayed processing in each panel. The Bonferroni threshold for this experiment was 0.005 (10 comparisons) and all statistics which reached significance below this are highlighted in bold. NfL and tTau concentrations were natural log-transformed. *GFAP* glial fibrillary acidic protein; *Haem* haemoglobin; *HD* Huntington’s disease; *NfL* neurofilament light; *SPMS* secondary progressive multiple sclerosis; *tTau* total tau

After confirming the stability of NfL following a three-day delay in processing, we proceeded to extend this delay and examined the impact of a seven-day processing delay on analyte concentrations in plasma (B.1.b) and serum (B.2.b) from healthy controls, HD, and MS participants. Total protein concentrations remained variable with seven days delayed processing in serum (Venous: *r = *0.389, *R*^2^ = 0.151, 95% CI 0.002–0.675, *p = *0.0495, Fig. 4A, B; Capillary: *r = *0.740, *R*^2^ = 0.548, 95% CI 0.494–0.876, *p < *0.0001, Fig. 4C, D). Haemoglobin concentrations increased in samples processed with a seven-day delay compared to those processed on the day of collection for both venous (Fig. 4F, G; MD = 5.1 mg/mL, 95% CI 4.8–5.4, *p < *0.0001, Supplementary Fig. 9b) and capillary (Fig. 4H, I; MD = 4.4 mg/mL, 95% CI 3.9–4.8, *p < *0.0001, Supplementary Fig. 9a) serum samples.

Serum NfL concentrations showed robust positive correlations when samples were processed on the day of collection versus seven days later for both venous (*r = *0.986, *R*^2^ = 0.973, 95% CI 0.969–0.994, *p < *0.0001, Fig. 4K, L) and capillary (*r = *0.991, *R*^2^ = 0.981, 95% CI 0.979–0.996, *p < *0.0001, Fig. 4M, N) samples. The Bland–Altman analysis confirmed strong agreement across the NfL concentrations measured in both serum and plasma between zero- and seven-day delay processed samples (Supplementary Fig. 10i–l). This stability was not reflected in any of the other analytes. GFAP concentrations declined with seven days of delayed processing in both venous (Fig. 4Q, MD = 62.8 pg/mL, 95%CI 46.3–79.4, *p < *0.0001, Supplementary Fig. 9d) and capillary (Fig. 4S, MD = 39.9 pg/mL, 95% CI 27.3–52.4, *p < *0.0001, Supplementary Fig. 9c) serum samples. Concentrations of plasma tTau remained highly variable in serum venous (*r = *0.346, *R*^2^ = 0.120, 95% CI − 0.047 to 0.647, *p = *0.0831, Fig. 4U, V) and capillary (*r = *0.399, *R*^2^ = 0.159, 95% CI 0.013–0.681, *p = *0.0437, Fig. 4W, X) samples. Analyte concentrations in plasma after a seven-day delay in processing compared to day zero concentrations were very similar to results for serum (Supplementary Fig. 11, 12).

### Capillary NfL shows the same clinical disease group differences as venous NfL in the multi-disease discovery cohort

**Fig. 5:**
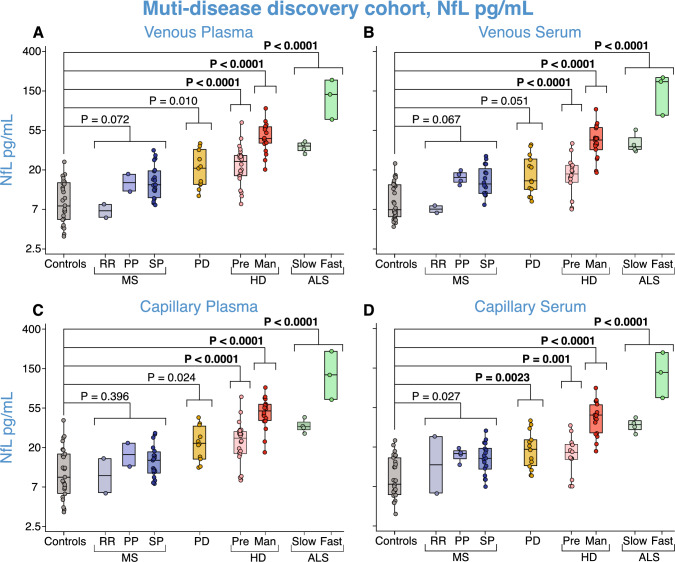
NfL group comparisons for all day-zero samples in the multi-disease discovery cohort. Disease group comparison of NfL concentrations in the multi-disease discovery cohort from **A** venous plasma, **B** venous serum, **C** capillary plasma, and **D** capillary serum from all baseline data for healthy controls, PPMS, RRMS, SPMS, PD, Pre-HD, manifest-HD, ALS-slow and ALS-fast patients. *p* values were generated from multiple linear regressions. The Bonferroni threshold for this experiment was 0.0025 (20 comparisons), and all statistics which reached significance below this are highlighted in bold. NfL values are natural log-transformed. *ALS* amyotrophic lateral sclerosis; *HD* Huntington’s disease; *Man* manifest; *MS* multiple sclerosis; *NfL* neurofilament light; *PD* Parkinson’s disease; *PP* primary progressive; *Pre* premanifest; *RR* relapsing–remitting; *SP* secondary progressive

As an exploratory analysis, we combined samples processed on the day of collection from all experiments to compare the ability of NfL concentrations from each sample and collection type to replicate disease difference patterns from venepuncture in the multi-disease discovery cohort (Fig. 5). The same pattern of disease group differences was seen for all collection and sample types. Compared to controls, pre-HD, manifest-HD, and ALS groups showed significantly elevated NfL concentrations for all collection types when controlling for age with similar MD (all *p* values < 0.0001, Fig. 5A–D, Supplementary Table 8). For each collection type, MS groups showed no significant difference in NfL concentrations compared to healthy controls apart from in capillary serum samples which showed significantly higher NfL levels but did not survive Bonferroni correction (MD = 5.81 pg/mL, 95% CI 0.37–0.6, *p = *0.027, Fig. 5D) and MD was similar (Supplementary Table 8). PD had significantly elevated NfL levels from healthy controls in venous plasma, capillary plasma and serum samples but only capillary serum survived Bonferroni correction (Venous plasma: MD = 11.26 pg/mL, 95% CI 0.11–0.83, *p = *0.010, Fig. 5A; Capillary plasma: MD = 11.65 pg/mL, 95% CI 0.06–0.88, *p = *0.024, Fig. 5C; Capillary serum: MD = 9.13, 95% CI 0.06–0.76, *p = *0.0023, Fig. 5D). There were no significant differences between disease groups and healthy controls for GFAP measures and this was consistent across sample and collection types (Supplementary Fig. 13, Supplementary Table 9).

### Consistency between NfL concentrations in venous and capillary blood samples replicated in the HD confirmatory cohort

**Fig. 6:**
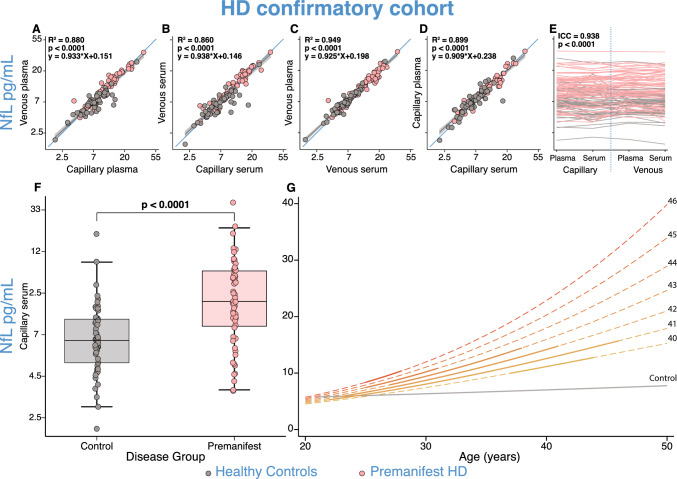
Replicating NfL consistency and disease phenotype across sample type in HD confirmatory cohort. NfL analyte concentrations across different collection methods and sample types from the HD confirmatory cohort (**A**–**E**) in healthy controls and pre-HD individuals. Blue lines represent the line of equality (*y* = *x*). Grey lines represent the linear regression fit of the data, with 95% confidence interval shaded regions. *R*^2^ and p-values were generated from regression models comparing the two collection types in each panel. The Bonferroni threshold for this experiment was 0.0125 (4 comparisons) and all statistics which reached significance below this are highlighted in bold. NfL concentrations were natural log-transformed. **F** Disease group differences between healthy controls and pre-HD in the HD confirmatory cohort from capillary serum samples. *p* values were generated from multiple linear regressions. Boxes show first and third quartiles, the central band shows the median, and the whiskers show data within 1.5 IQR of the median. The Bonferroni threshold for this experiment was 0.025 (2 comparisons) and statistics which reached significance below this are highlighted in bold. NfL values were natural log-transformed. **G** Modelling CAG-Age-NfL relationships to show associations between NfL, age, and CAG repeat count using capillary serum samples. Solid lines were produced from our observations using a multiple linear regression model accounting for the main effects of age, CAG, the quadratic effect of age, and the interaction between age and CAG; dashed lines are predictions outside the range of our observations. Separate figures with individual data points for each individual CAG repeat count are provided in Supplementary Fig. 16. *HD* Huntington’s disease; *NfL* neurofilament light

Similar to the multi-disease discovery cohort, in the HD confirmatory cohort NfL concentrations were strongly correlated between venous and capillary samples in both plasma (*r = *0.938, *R*^2^ = 0.880, 95% CI 2.49–2.6, *p < *0.0001; Fig. 6A) and serum (*r = *0.927, *R*^2^ = 0.860, 95% CI 2.45–2.59, *p < *0.0001; Fig. 6B), and plasma and serum NfL values were equivalent in both collection types (Venous: *r = *0.974, *R*^2^ = 0.949, 95% CI 2.62–2.67, *p < *0.0001; Fig. 6C; Capillary: *r = *0.948, *R*^2^ = 0.899, 95% CI 2.52–2.62, *p < *0.0001; Fig. 6D). Total protein, haemoglobin, GFAP, and tTau concentrations across sample and collection type were very similar to the multi-disease discovery cohort (Supplementary Fig. 14).

Capillary serum and plasma NfL concentrations between pre-HD and healthy controls replicated venous blood disease group differences, previously reported in the literature (Serum: Fig. 6F; Plasma: Supplementary Fig. 15a) [7, 8, 14, 30]. Compared to controls, pre-HD individuals showed significantly elevated capillary serum NfL concentrations when controlling for age (MD = 1.55 pg/mL, 95% CI 1.3–1.77, *p < *0.0001; Fig. 6F; Supplementary Table 10), also replicated in capillary plasma and venous samples (Supplementary Table 10). Furthermore, by measuring NfL concentrations in capillary samples, we successfully replicated models based on venous plasma, demonstrating a CAG-dependent increase in NfL levels in participants with HD (Serum: Fig. 6G, Supplementary Table 11, 12, Supplementary Fig. 16; Plasma: Supplementary Fig. 15b, Supplementary Table 13, 14, Supplementary Fig. 17) [7, 14]. In healthy controls, the relationship between capillary serum NfL and age was approximately linear (slope 0.01 log pg/mL per year [SE 0.0042], *p = *0.017; Supplementary Table 11). In HD patients, we recapitulated the non-linear CAG-dependent relationship with age, showing that NfL concentrations in capillary serum increased as CAG repeat length increased (Fig. 6G; Supplementary Table 12; Supplementary Fig. 16). CAG-dependent relationships were also replicated using capillary plasma samples (Supplementary Table 13, 14; Supplementary Fig. 17).

## Discussion

In this study, we investigated the potential of an optimised finger-prick blood collection approach for remote quantification of NfL in a multi-disease discovery cohort of four different neurological conditions (HD, MS, ALS, PD) via a series of experiments that directly compared the impact of the collection method, processed sample type, and delayed processing on analyte concentrations. Our findings demonstrated that NfL and GFAP concentrations were equivalent whether measured from capillary or venous blood, and whether processed into serum or plasma. NfL levels were able to withstand delayed processing of up to seven days. In a confirmatory HD cohort, we further replicated that collection method and sample processing type had no significant impact on NfL and GFAP concentrations. We also replicated previously published cross-sectional HD-related findings from venous blood with our finger-prick collected capillary serum, providing evidence to support the clinical utility of this method [7, 8, 14].

The methodologies employed in this study represent, to the best of our knowledge, the first use of a capillary finger-prick blood collection approach for biomarker assessment in neurological disorders. Previous reports of alternative collection and processing methods for NfL quantification, such as DPS using Noviplex Plasma Prep cards, were performed using venous blood, and despite being highly correlated, DPS NfL values were reported to be approximately four times lower than standard venous NfL concentrations [19, 20]. Here, we report regression fits and data points for each condition comparison of NfL concentrations to lie on the line of equality in both the multi-disease discovery cohort and HD confirmatory cohort, an indication of equivalence.

Despite variations in haemolysis produced by each method, concentrations of NfL and GFAP remained consistent in both venous and capillary samples irrespective of processed sample type; this mirrors previous studies that have investigated the translatability of NfL and GFAP between serum and plasma samples [32–34], although some studies have reported serum NfL to be higher than plasma NfL [35, 36]. This marks an advancement in our understanding of the feasibility and accuracy of this minimally invasive approach to quantify a key biomarker of neuronal injury outside the clinic environment. The strong agreement that GFAP concentrations also demonstrated between venous and capillary blood is promising, as it supports the development of additional blood-based biomarkers for remote quantification.

The robust agreement between sample and collection type for NfL and GFAP was not replicated in other analytes: there was high variability in tTau levels between capillary and venous blood, as well as between serum and plasma in each collection method. This is in line with previous reports that found no overall correlation between plasma and serum tTau levels [32, 37]. Consequently, considerations regarding the choice of collection method and sample type (venepuncture, finger-prick, serum, or plasma) need to be further assessed for blood tTau measurements.

Our results demonstrate that NfL exhibits stability up to seven days delayed processing in both venous and capillary samples. This replicates the stability of NfL in venous blood presented in previous studies [6, 38] and further demonstrates its stability in capillary blood samples. GFAP concentrations declined after a seven-day delay in processing, with signs of reduction even at a three-day delay. Given the persistence of strong correlations despite these reductions in GFAP concentrations, the impact of delayed processing on GFAP may be consistent and quantifiable. If this can be quantified, it could support the use of a conversion factor to account for the impact of delayed processing. This is outside the scope of the current study but may warrant further study for disease conditions where GFAP is a strong candidate biomarker.

By quantifying total protein and haemoglobin, we were able to gain insight into the generalised impact of collection, sample type, and processing delay on proteins in the blood that could present confounding effects on biomarker quantification. As expected, haemoglobin increased with increasing delay in sample processing, consistent with the increased haemolysis visibly seen when processing these samples. The subtle increases in total protein after three days and lack of change after seven days of delayed processing could be due to the increased haemolysis causing subtle increases in total protein concentrations outweighing any protein degradation [39, 40]. We would expect haemolysis to plateau with further increases in delay interval and for total protein to begin to decrease as proteins degrade. These effects should be investigated further to better understand the dynamics at play.

The delay interval investigated in this study spanned seven days; the longest delay interval studied for venous blood NfL is eight days where the analyte was shown to be stable [6]. The potential of a remote sample collection for NfL quantification could facilitate a wide-scale international study of this biomarker. The complexities with international shipping increase the risk of longer processing delays where delivery may take more than a week. Therefore, further studies should consider investigations extending beyond this duration in capillary samples. We are currently collecting matched in-person samples processed after delay periods of 10-, 12-, and 15-days, which will allow us to better understand the degradation profile of NfL and help identify a threshold beyond which stability may be compromised.

Both NfL and GFAP have broad relevance across multiple neurological disorders as blood-based biomarkers. We have reproduced previous findings that NfL is significantly elevated in HD [7], PD [41], and ALS [13], compared to controls using capillary blood samples. The HD confirmatory cohort included participants recruited from HD-YAS, a longitudinal study aimed to characterise and identify the earliest manifestations of HD pathology [14, 29]. In capillary blood, we successfully replicated previous findings that venous plasma NfL concentrations were significantly elevated in pre-HD individuals compared to matched controls, and confirmed the non-linear CAG-dependent relationship between age and NfL levels [7, 8, 14], suggesting that our remote sampling method for NfL measurement may be useful in these disease populations.

Blood NfL could have several applications in therapeutic development and clinical management in neurology. NfL has been proposed as a potential surrogate endpoint indicating neuroprotection for multiple neurological disorders (e.g., MS [42], ALS [43], and HD [7]); its utility as a safety biomarker for neurotoxicity is also being considered [44, 45]; and it may be further utilised for enrichment and stratification of presymptomatic populations to facilitate prevention trial design [46]. However, before blood NfL can be validated for these clinical applications, there needs to be deep characterisation of its natural fluctuations throughout the progression of each disease in question. Remote collection offers the potential to facilitate frequent short-interval sampling and real-world monitoring of patients. Not only would this reduce the patient burden of in-person visits, it may also expedite indications of drug efficacy, toxicity, or clinical progression due to the increased feasibility of short-interval sampling. Monitoring NfL levels throughout disease progression may aid preclinical staging and inform clinical care decisions and planning. Large-scale, prospective and longitudinal remote sampling for NfL quantification with more detailed clinical phenotyping are now needed to fully characterise blood NfL across the natural history of each neurological disorder. Through HD-YAS, we are actively employing remote finger-prick blood collection kits, enabling participants to provide frequent bi-monthly samples from home for NfL analysis. This short-interval sampling data will enable a deep characterisation of blood NfL dynamics within this early-stage cohort, including insight into the divergence point of NfL levels in people with HD compared to controls.

Our study has several limitations: Firstly, accurately simulating blood sample shipment conditions in controlled experiments is extremely difficult. In our study, samples were left for three or seven days on a shaker at room temperature in our laboratory. This did not simulate the significant temperature fluctuations that sample kits may endure whilst being shipped from the patients’ homes to the laboratory. The temperature that parcels are exposed to varies greatly depending on the mode of transportation, weather conditions, duration of transport and specific handling procedures from the courier service. Previous data have shown that NfL can sustain multiple freeze–thaw cycles [21, 22], and up to 24 h at 37 °C incubation [19, 20]. There is no evidence to our knowledge that confirms that it can sustain higher temperatures. Furthermore, a shaker cannot replicate the unpredictable mechanical stresses that the samples go through during shipping. Indeed, samples may fall during transport or could be left still for many hours at a time. It may be important to repeat our experiment leaving the samples still on the bench, inducing random mechanical stresses, increasing delay intervals, and employing a varied temperature protocol to determine whether these events affect NfL stability, both separately and simultaneously.

Second, although we showed that capillary NfL reproduced similar disease group differences from previously reported cohort studies, our study was not powered for this post-hoc analysis. At-home collections will need to be set up and assessed for each disease aiming to clinically validate remote NfL quantifications for its population. Third, as this was a technical validation of the finger-prick approach, collections were performed by our team. We have not yet shown the feasibility of patients performing self-sampling at home without a health professional guiding them. However, we have already established ongoing remote collections with over 100 individuals (pre-HD and matched healthy controls) actively collecting blood in their own home and returning the samples by post to our laboratory every two months. We are planning other studies in clinical populations to address this further. Finally, for tTau, the recommended sample matrix is EDTA plasma [47], which was not available in our study. We cannot exclude that remote collection of capillary EDTA plasma would produce better results.

## Conclusions

In conclusion, this study has shown that the finger-prick sample collection approach for NfL measurement may be reliable and useful in several disease populations. According to our results, NfL can be measured from capillary plasma and serum seven days after remote collection, with equivalent concentrations to the current gold standard of plasma and serum collection (in-clinic venepuncture with same-day blood processing and storage). A crucial element in accelerating patient-centred healthcare involves the capability to remotely collect blood samples with the same quality as traditional phlebotomy, facilitating regular monitoring of disease progression and testing of drug responses to therapeutic intervention. With further validation, we believe this approach could be used for remote monitoring of NfL levels in neurological disease populations, with higher sampling frequency and geographical patient outreach than in-clinic blood collections could accommodate. The applications for NfL across the spectrum of neurological disorders emphasise the widespread translatability of these methods, which have the potential to transform disease monitoring, prognosis, and therapeutic development within clinical trials and practice.

## Supplementary Information

Below is the link to the electronic supplementary material.

Supplementary file1 (PDF 8458 KB)

## Data Availability

The datasets used and/or analysed during the current study are available from the corresponding author upon reasonable request.
