# Supplementary material for: Evaluating finger-prick blood collection for remote quantification of neurofilament light in neurological diseases
Source: J Neurol. 2025 Jul 10;272(8):501. doi: 10.1007/s00415-025-13232-8 (PMC12245952; doi:10.1007/s00415-025-13232-8)
Supplement: Supplementary file 1 — Supplementary file1 (PDF 8458 KB) [file 415_2025_13232_MOESM1_ESM.pdf]

Evaluating finger-prick blood collection for remote quantification of neurofilament light in neurological diseases

Journal of Neurology

Supplementary tables and figures

Annabelle Coleman[1†]; Alexiane Touzé[1†]; Mena Farag[1]; Marta Pengo[1,2,3]; Michael J Murphy[1]; Yara Hassan[1]; Olivia Thackeray[1]; Kate Fayer[1]; Sophie Field[1]; Mitsuko Nakajima[1]; Elizabeth L Broom[1]; Nicola Z Hobbs[1]; Brook Huxford[4]; Natalie Donkor[4]; Ellen Camboe[4,5]; Kamalesh C Dey[4,5]; Alexandra Zirra[4,5]; Aisha Ahmed[6,7]; Ana Rita Gameiro Costa[6-8]; Harriet Sorrell[6,7,9]; Luca Zampedri[6,7]; Vittoria Lombardi[6,7]; Charles Wade[10]; Sean Mangion[10]; Batoul Fneich[10]; Amanda Heslegrave[11]; Henrik Zetterberg[11-15]; Rachael Scahill[1]; Alastair Noyce[4,5]; Andrea Malaspina[6,7]; Jeremy Chataway[10,16]; Sarah J Tabrizi[1]; Lauren M Byrne[1*].

† These authors contributed equally as first authors of this study.

*** Corresponding author:**

Dr Lauren M Byrne, lauren.byrne.14@ucl.ac.uk, +447709524351

UCL Huntington’s Disease, UCL Queen Square Institute of Neurology, Queen Square, London, WC1N 3BG, UK

Table of Contents

**** Corresponding author:*** 1

Fig. 1 – Pilot NfL concentrations in venous versus capillary plasma 4

Fig. 2 – A.0.0 total protein and haemoglobin in the multi-disease discovery cohort 5

Fig. 3 – A.0.0 paired t-tests in multi-disease discovery cohort 6

Fig. 4 – A.0.0 Bland-Altman plots in multi-disease discovery cohort 7

Fig. 5 – B.2.a serum paired t-tests in the multi-disease discovery cohort 8

Fig 6 – B.1.a and B.2.a 3-day delay Bland Altman plots in the multi-disease discovery cohort 9

Fig. 7 – B.1.a plasma 3-day delay results in multi-disease discovery cohort 10

Fig. 8 – B.1.a plasma paired t-tests in multi-disease discovery cohort 11

Fig. 9 – B.2.b serum paired t-tests in the multi-disease discovery cohort 13

Fig. 10 – B.1.b and B.2.b 7-day delay Bland Altman plots in the multi-disease discovery cohort 14

Fig. 11 – B.1.b plasma 7-day delay results in multi-disease discovery cohort 15

Fig. 12 – B.1.b plasma paired t-tests in the multi-disease discovery cohort 16

Fig. 13 – GFAP group comparisons for all baseline data in multi-disease discovery cohort 17

Fig. 14 – A.0.0 results replicated in HD confirmatory cohort 18

Fig. 15 – Capillary plasma NfL group comparisons in the HD confirmatory cohort and CAG-Age-NfL plot 19

Fig. 16 – Individual CAG plots for CAG-NfL-Age models from capillary serum samples 20

Fig. 17 – Individual CAG plots for CAG-NfL-Age models from capillary plasma samples 21

Table 1 – Technical variation 22

Table 2 – Group demographics for the multi-disease discovery cohort and confirmatory cohort 23

Tables 3 – 7: Summary statistics for each analyte in the multi-disease discovery cohort and HD confirmatory cohort 24

Table 8 – Mean differences for multi-disease discovery cohort for NfL 29

Table 9 – Mean differences for multi-disease discovery cohort for GFAP 30

Table 10: Mean differences for HD confirmatory cohort for NfL 31

Table 11 – 12: Relationships between capillary serum NfL, age and CAG repeat in the HD confirmatory cohort 32

Table 13 – 14: Relationships between capillary plasma NfL, age and CAG repeat in the HD confirmatory cohort 33

Fig. 1 – Pilot NfL concentrations in venous versus capillary plasma

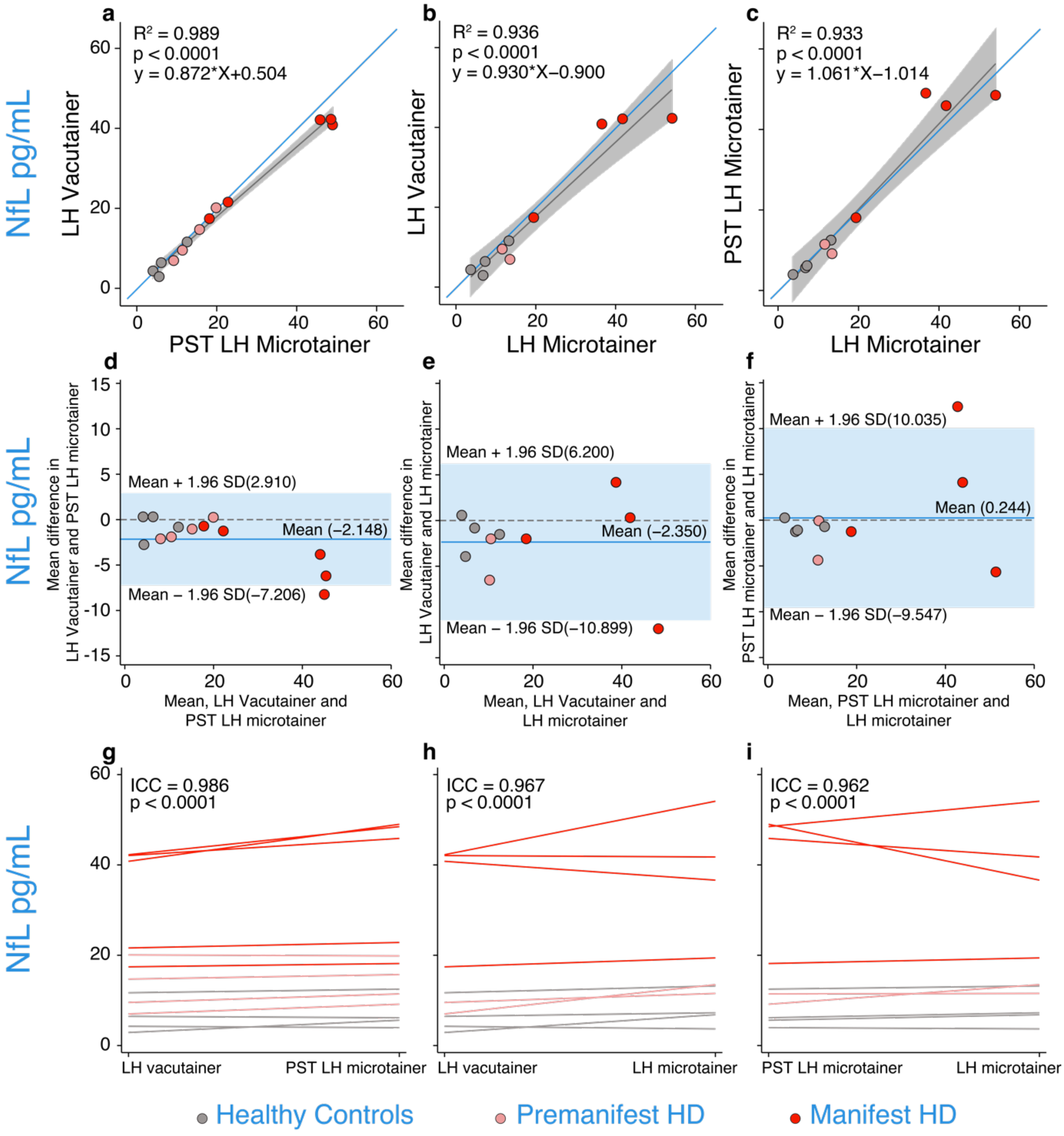

***Fig. 1** Pilot venous versus capillary tube type comparison data showed our finger-prick method is closer to y=x than previously reported DPS methods (21, 22). Panels a-c are scatter plots of NfL concentrations measured from a) LH vacutainer versus PST LH microtainer, b) LH vacutainer versus LH microtainer, and c) PST LH microtainer versus LH microtainer. Blue lines represent y=x. Grey lines represent the linear regression fit of the data. R^2 and P values are generated from regression models comparing the two collection types in each panel. Panels d-f show Bland-Altman plots for agreement between tube-type for NfL measurements with the 95% limits of agreement represented by the limits of the light blue shaded region; dark blue lines represent the mean; dashed line represents y=0; d) LH vacutainer versus PST LH microtainer, e) LH vacutainer versus LH microtainer, f) PST LH microtainer versus LH microtainer. Panels g-i show spaghetti plots to represent the reliability of NfL measurements between g) PST LH microtainer versus LH microtainer, h) LH vacutainer versus LH microtainer, and i) LH microtainer versus PST LH microtainer. HD, Huntington's disease; LH, Lithium Heparin; NfL, Neurofilament light; PST, Plasma separating tube*

Fig. 2 – A.0.0 total protein and haemoglobin in the multi-disease discovery cohort

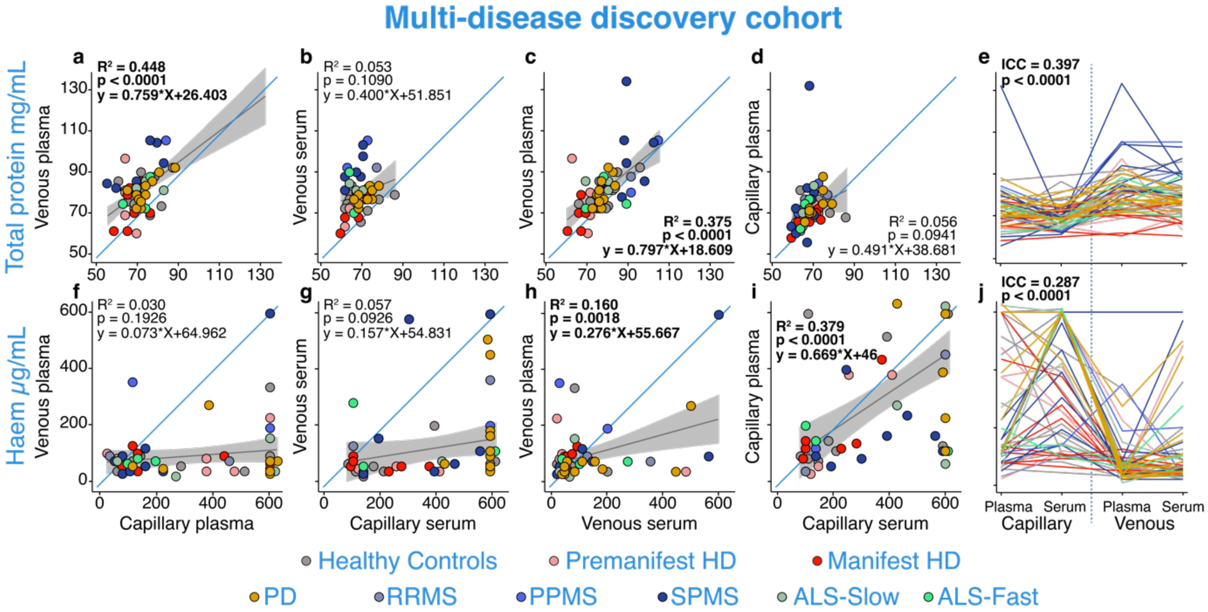

Fig. 2 *Analyte concentrations across different collection methods and sample types in the multi-disease discovery cohort from experiment A.0.0 for total protein (a-e), and haemoglobin (f-j) in healthy controls, pre-HD, manifest-HD, PD, RRMS, PPMS, SPMS, ALS-slow, ALS-fast. Blue lines represent y=x. Grey lines represent the linear regression fit of the data with 95% confidence interval shaded regions.* R^2 *and p-values were generated from regression models comparing the two collection types in each panel. The Bonferroni threshold for this experiment was 0.00625 (8 comparisons) and all statistics which reached significance below this are highlighted in bold. ALS, amyotrophic lateral sclerosis; Haem, haemoglobin; HD, Huntington's disease; PD, Parkinson's disease; PPMS, primary progressive multiple sclerosis; RRMS, relapsing-remitting multiple sclerosis; SPMS, secondary progressive multiple sclerosis; tTau, total tau*

Fig. 3 – A.0.0 paired t-tests in multi-disease discovery cohort

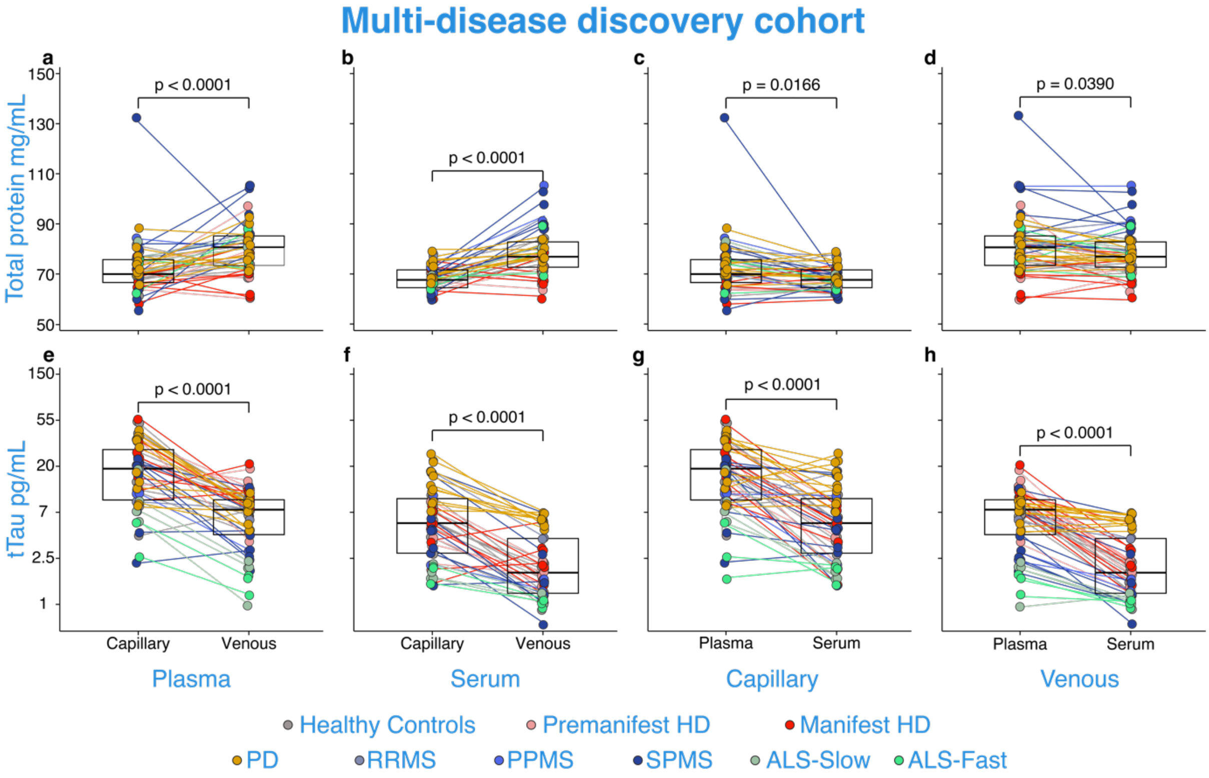

Fig. 3 *Concentrations of total protein (a-d), and tTau (e-h) from capillary and venous plasma and serum from experiment A.0.0 in the multi-disease discovery cohort. p-values were generated from paired t-tests. Boxes represent the interquartile range with bold black horizontal line indicating the median. tTau concentrations are natural log transformed. ALS, amyotrophic lateral sclerosis; Haem, haemoglobin; HD, Huntington's disease; PD, Parkinson's disease; PPMS, primary progressive multiple sclerosis; RRMS, relapsing-remitting multiple sclerosis; SPMS, secondary progressive multiple sclerosis; tTau, total tau*

Fig. 4 – A.0.0 Bland-Altman plots in multi-disease discovery cohort

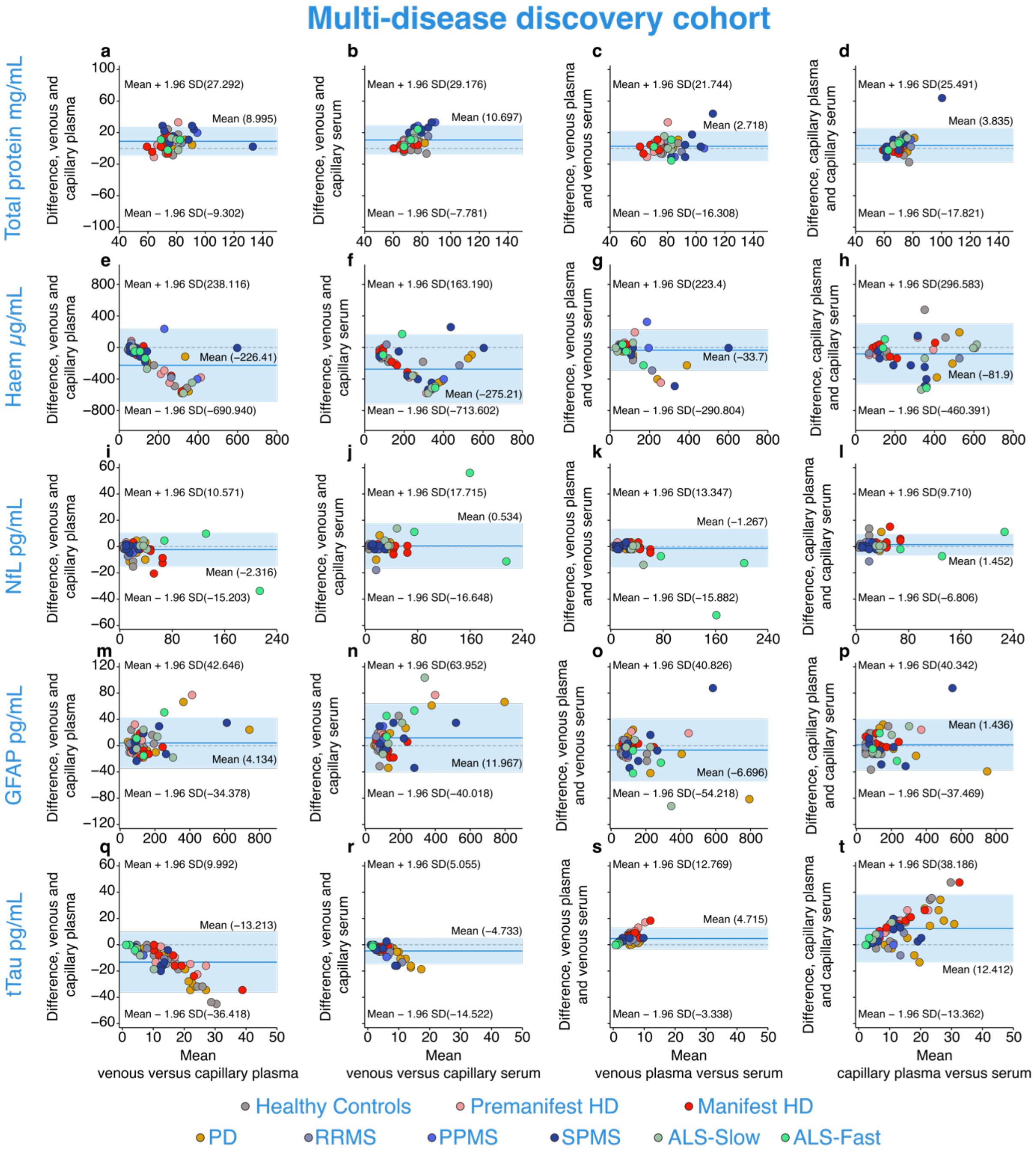

Fig. 4 *Bland-Altman plots across different collection methods and sample types in the multi-disease discovery cohort from experiment A.0.0 for total protein (a-d), haemoglobin (e-h), NfL (i-l), GFAP (m-p), and tTau (q-t) in healthy controls, pre-HD, manifest-HD, PD, RRMS, PPMS, SPMS, ALS-slow, ALS-fast. 95% limits of agreement are represented by the limits of the light blue shaded region, dark blue lines represent the mean, and the dashed line represents y=0 i.e. no difference. ALS, amyotrophic lateral sclerosis; GFAP, glial fibrillary acidic protein; Haem, haemoglobin; HD, Huntington's disease; NfL, neurofilament light; PD, Parkinson's disease; PPMS, primary progressive multiple sclerosis; RRMS, relapsing-remitting multiple sclerosis; SPMS, secondary progressive multiple sclerosis; tTau, total tau*

Fig. 5 – B.2.a serum paired t-tests in the multi-disease discovery cohort

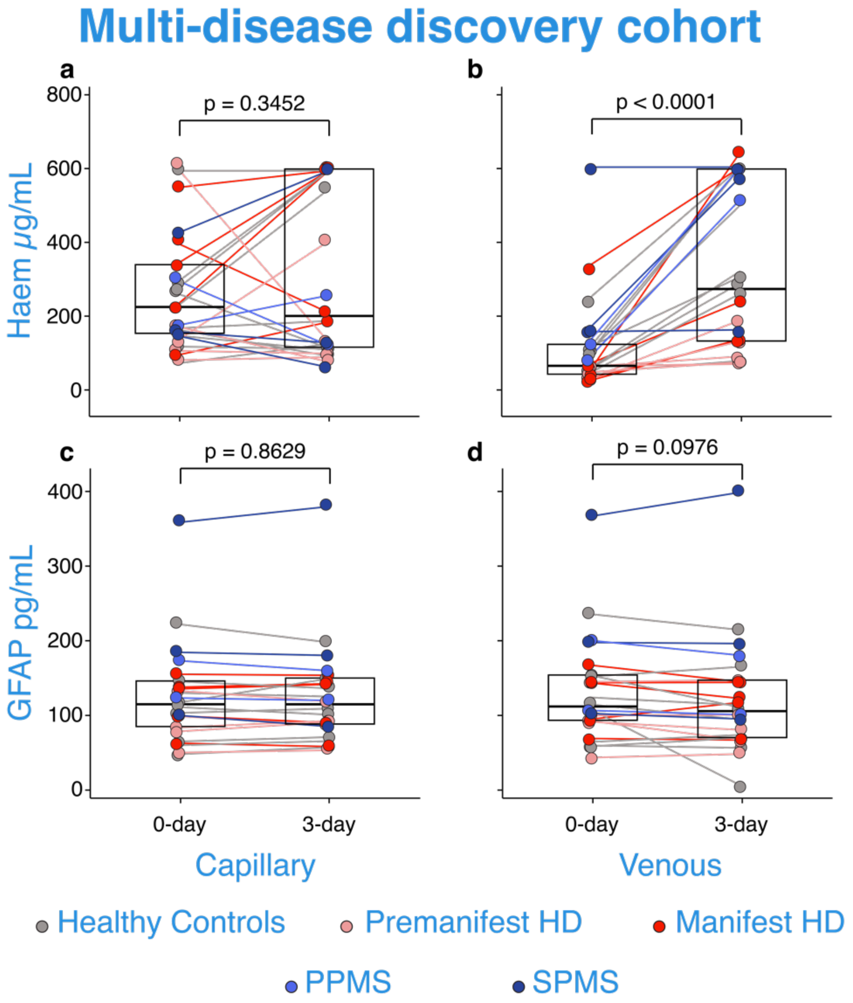

Fig. 5 *Concentrations of haemoglobin (a-b) and GFAP (c-d) in capillary and venous serum when processed on the day of collection and when processed three days later from experiment B.2.a in the multi-disease discovery cohort. p-values were generated from paired t-tests. Boxes represent the interquartile range with bold black horizontal line indicating the median. GFAP; glial fibrillary acidic protein; Haem, haemoglobin; HD, Huntington's disease; PPMS, primary progressive multiple sclerosis; SPMS, secondary progressive multiple sclerosis*

<u>Fig 6 – B.1.a and B.2.a 3-day delay Bland Altman plots in the multi-disease discovery cohort</u>

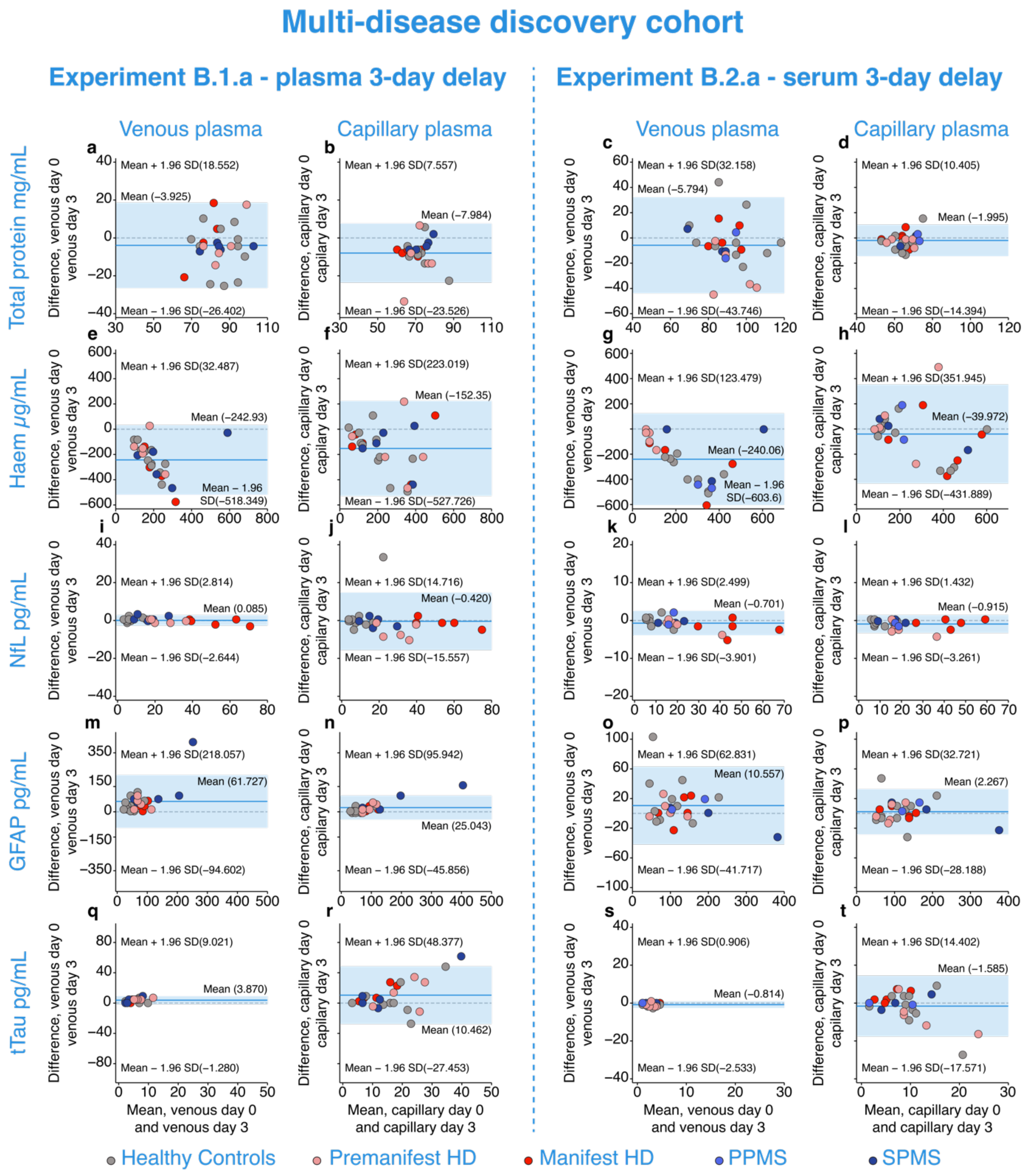

Fig 6 *Bland-Altman plots of mean differences of analyte concentrations in the multi-disease discovery cohort between zero-day and three-day delay in processing from experiment B.1.a and B.2.a for total protein (a-b), haemoglobin (e-f), NfL (i-j), GFAP (m-n), and tTau (q-r) and B.2.a for total protein (c-d), haemoglobin (g-h), NfL (k-l), GFAP (o-p), and tTau (s-t) in healthy controls, pre-HD, manifest-HD, PPMS, SPMS. 95% limits of agreement are represented by the limits of the light blue shaded region, dark blue lines represent the mean, and the dashed line represents y=0. GFAP, glial fibrillary acidic protein; Haem, haemoglobin; HD, Huntington's disease; NfL, neurofilament light; PPMS, primary progressive multiple sclerosis; SPMS, secondary progressive multiple sclerosis; tTau, total tau*

<u>Fig. 7 – B.1.a plasma 3-day delay results in multi-disease discovery cohort</u>

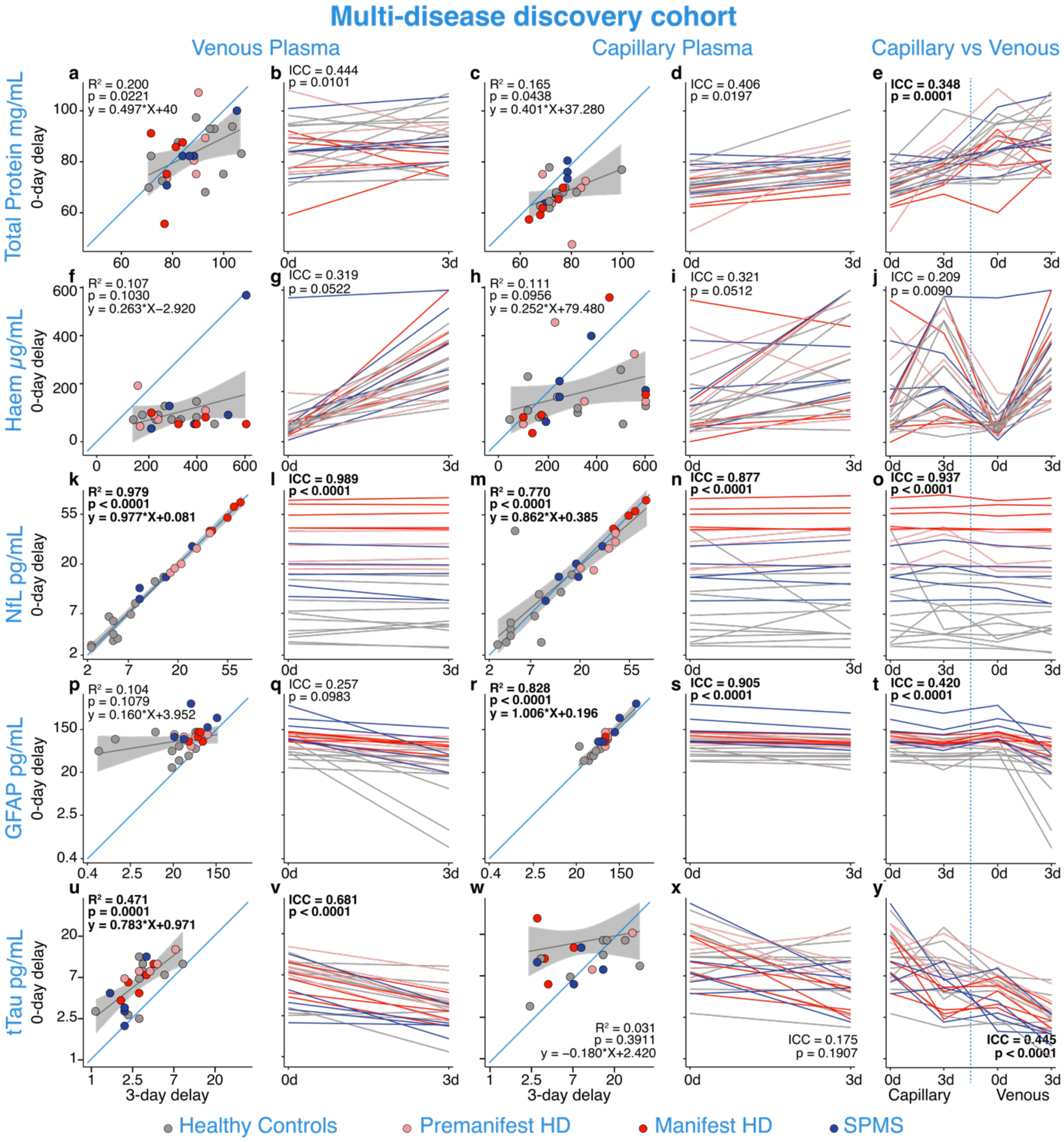

Fig. 7 *Analyte concentrations in plasma in the multi-disease discovery cohort after zero-day and three-day delay in processing from experiment B.1.a. for total protein (a-e), haemoglobin (f-j), NfL (k-o), GFAP (p-t), and tTau (u-y) from venous and capillary collections in healthy controls, pre-HD, manifest-HD, SPMS, Blue lines represent y=x. Grey lines represent the linear regression fit of the data with 95% confidence interval shaded regions.* R^2 *and p-values were generated from regression models comparing the impact of delayed processing in each panel. The Bonferroni threshold for this experiment was 0.005 (10 comparisons) and all statistics which reached significance below this are highlighted in bold. NfL, GFAP, and tTau concentrations were natural log-transformed. GFAP, glial fibrillary acidic protein; Haem, haemoglobin; HD, Huntington's disease; NfL, neurofilament light; SPMS, secondary progressive multiple sclerosis; tTau, total tau*

Fig. 8 – B.1.a plasma paired t-tests in multi-disease discovery cohort

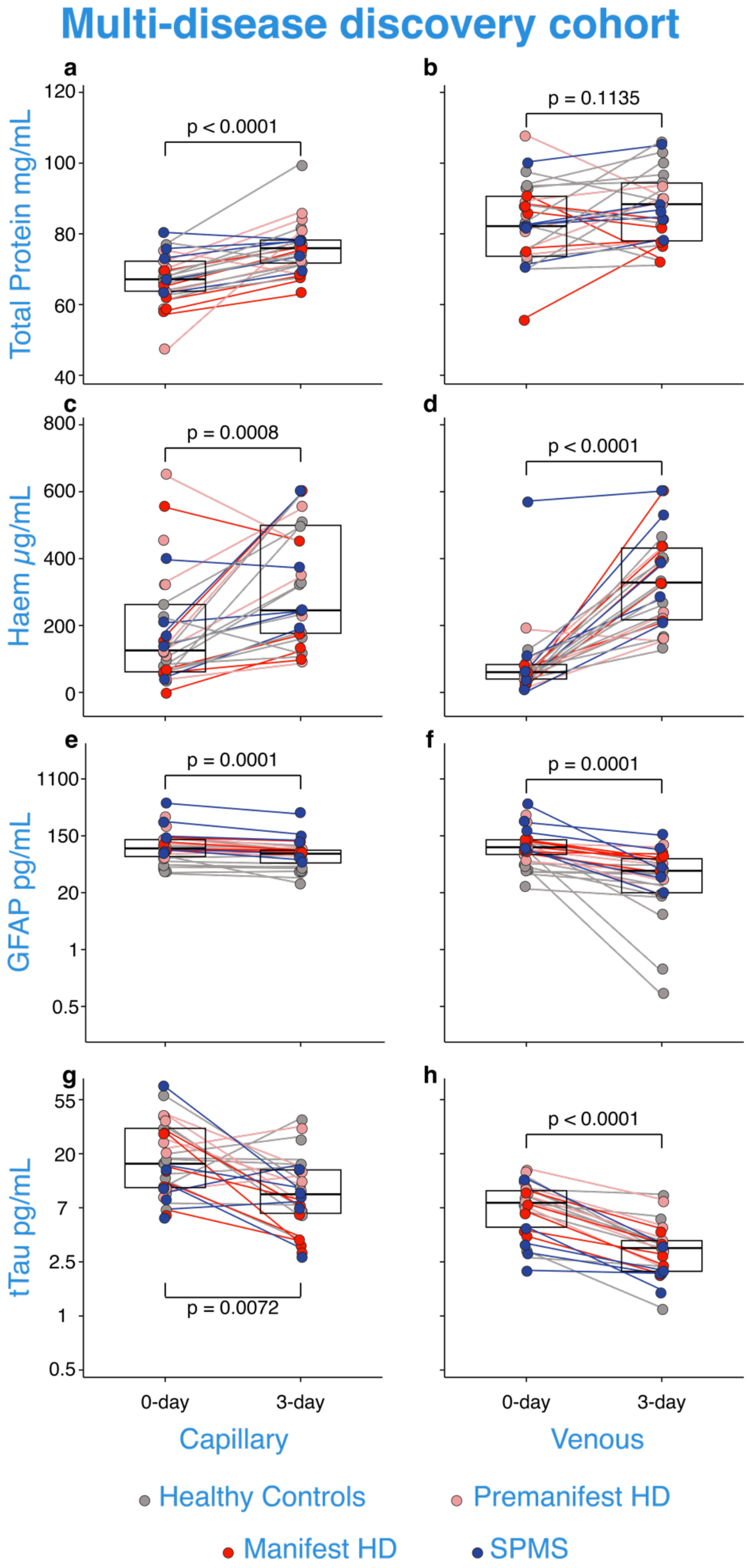

Fig. 8 *Concentrations of total protein (a-b), haemoglobin (c-d), GFAP (e-f), and tTau (g-h) in capillary and venous plasma when processed on the day of collection and when processed three days later from experiment B.1.a in the multi-disease discovery cohort. p-values were generated from paired t-tests. Boxes represent the interquartile range with bold black horizontal line indicating the median. GFAP and tTau concentrations are natural log transformed. GFAP, glial fibrillary acidic protein; Haem, haemoglobin; HD, Huntington's disease; SPMS, secondary progressive multiple sclerosis; tTau, total tau*

Fig. 9 – B.2.b serum paired t-tests in the multi-disease discovery cohort

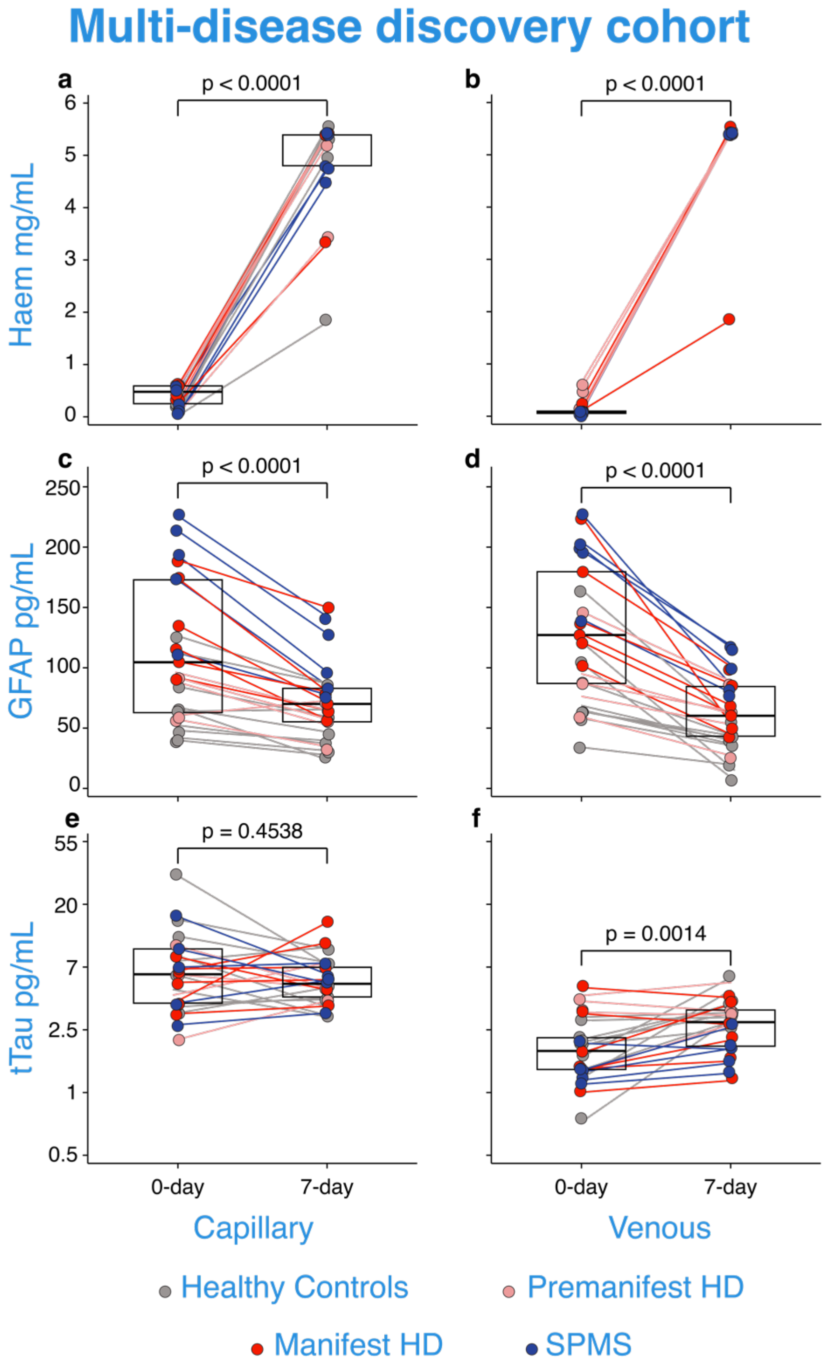

Fig. 9 *Concentrations of haemoglobin (a-b), GFAP (c-d), and tTau (e-f) from capillary and venous serum in the multi-disease discovery cohort when processed on the day of collection and when processed seven days later from experiment B.2.b. p-values were generated from paired t-tests. Boxes represent the interquartile range with bold black horizontal line indicating the median. tTau concentrations are natural log transformed. GFAP, glial fibrillary acidic protein; Haem, haemoglobin; HD, Huntington's disease; SPMS, secondary progressive multiple sclerosis; tTau, total tau*

Fig. 10 – B.1.b and B.2.b 7-day delay Bland Altman plots in the multi-disease discovery cohort

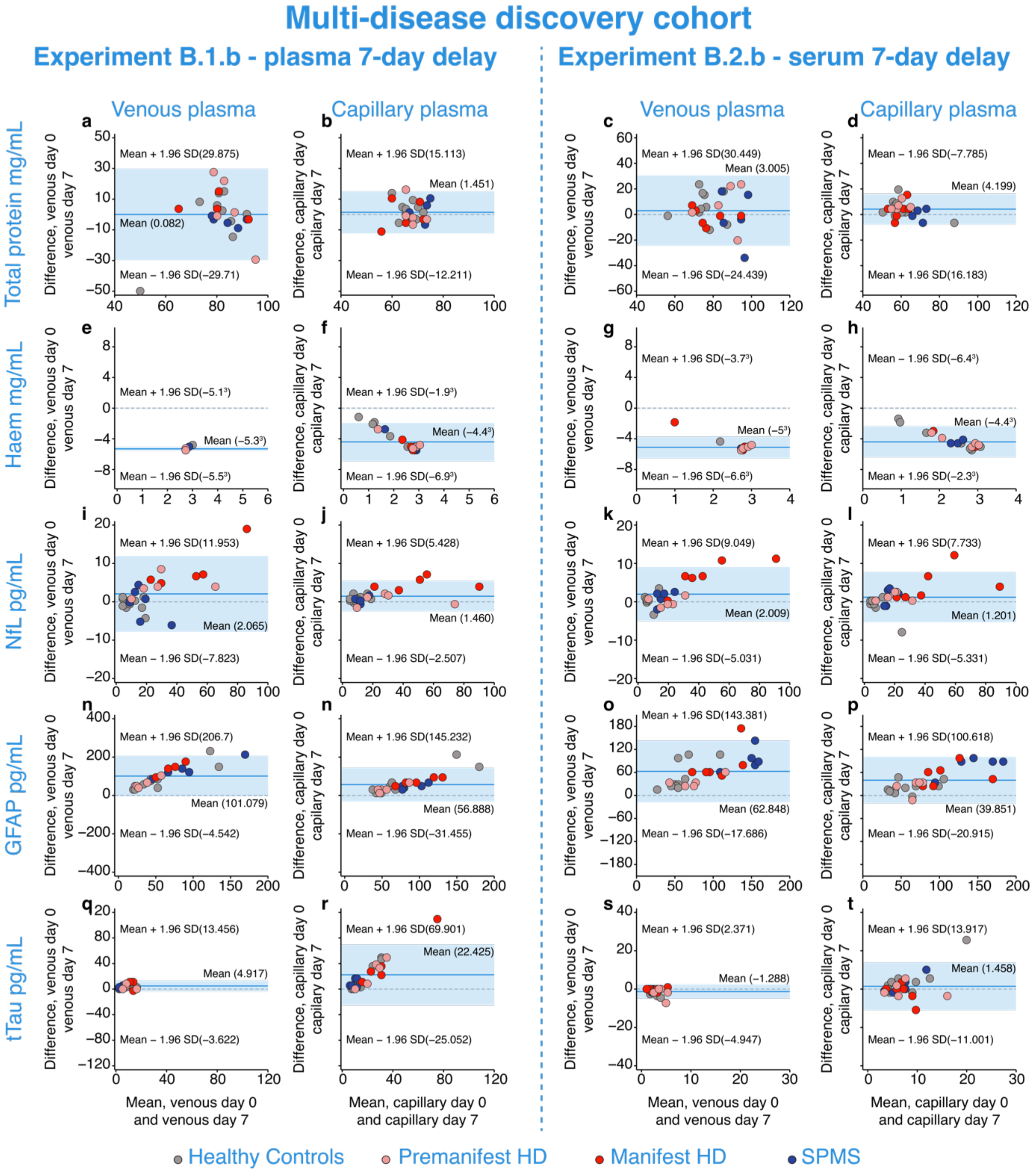

Fig. 10 *Bland-Altman plots of mean differences of analyte concentrations between zero-day and seven-day delay in processing from experiment B.1.b and B.2.b in the multi-disease discovery cohort for total protein (a-b), haemoglobin (e-f), NfL (i-j), GFAP (m-n), and tTau (q-r) and B.2.b for total protein (c-d), haemoglobin (g-h), NfL (k-l), GFAP (o-p), and tTau (s-t) in healthy controls, pre-HD, manifest-HD, PPMS, SPMS. 95% limits of agreement are represented by the limits of the light blue shaded region, dark blue lines represent the mean, and the dashed line represents y=0. NfL, GFAP (only B.1.b (m-n)), and tTau concentrations are natural log transformed. GFAP, glial fibrillary acidic protein; Haem, haemoglobin; HD, Huntington's disease; NfL, neurofilament light; SPMS, secondary progressive multiple sclerosis; tTau, total tau*

Fig. 11 – B.1.b plasma 7-day delay results in multi-disease discovery cohort

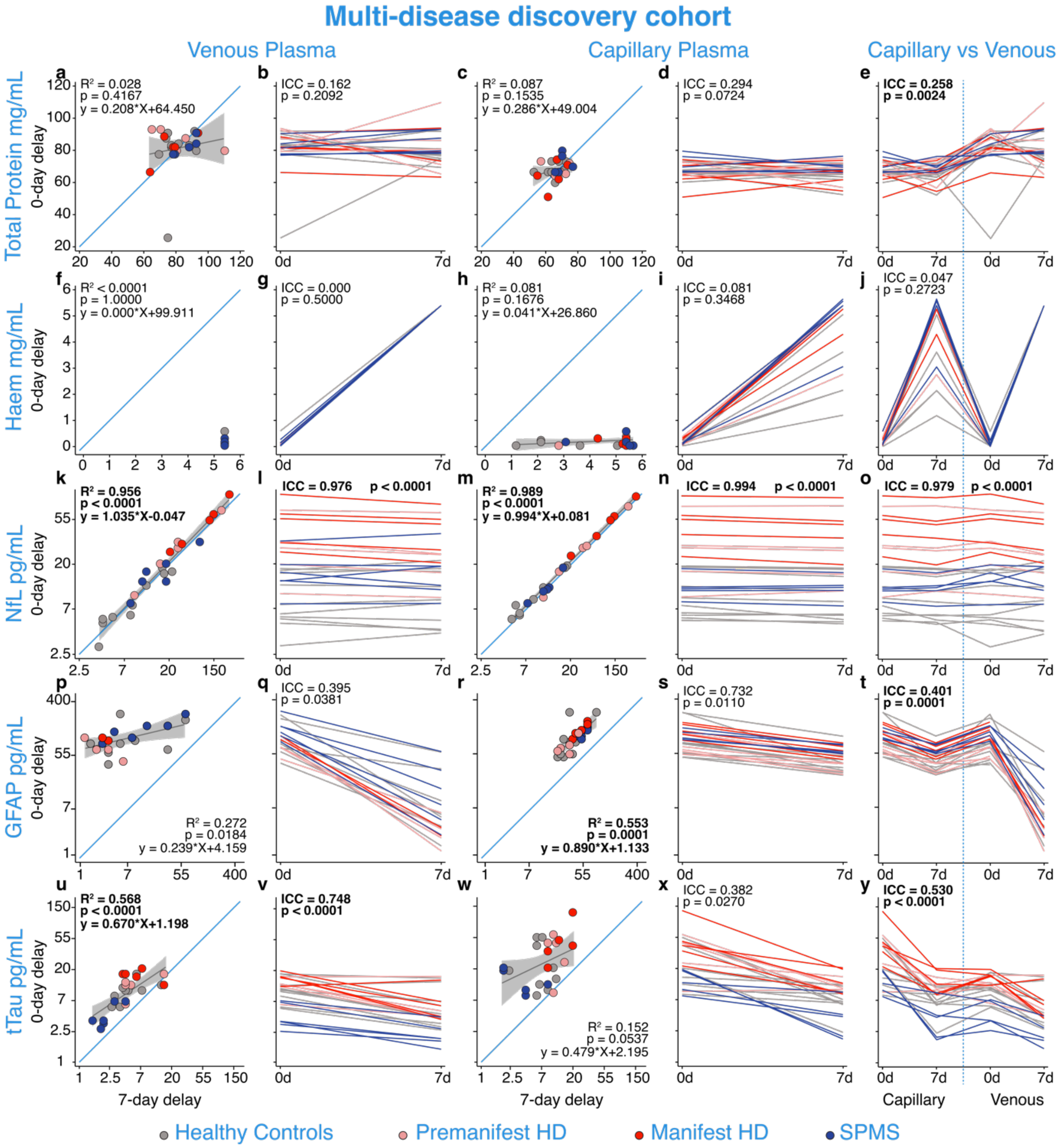

Fig. 11 *Analyte concentrations in plasma after zero-day and seven-day delay in processing from experiment B.1.b. in the multi-disease discovery cohort for total protein (a-e), haemoglobin (f-j), NfL (k-o), GFAP (p-t), and tTau (u-y) from venous and capillary plasma in healthy controls, pre-HD, manifest-HD, and SPMS. Blue lines represent y=x. Grey lines represent the linear regression fit of the data with 95% confidence interval shaded regions. R^2 and p-values were generated from regression models comparing the impact of delayed processing in each panel. The Bonferroni threshold for this experiment was 0.005 (10 comparisons) and all statistics which reached significance below this are highlighted in bold. NfL, GFAP, and tTau concentrations were natural log-transformed. GFAP, glial fibrillary acidic protein; Haem, haemoglobin; HD, Huntington's disease; NfL, neurofilament light; SPMS, secondary progressive multiple sclerosis; tTau, total tau*

Fig. 12 – B.1.b plasma paired t-tests in the multi-disease discovery cohort

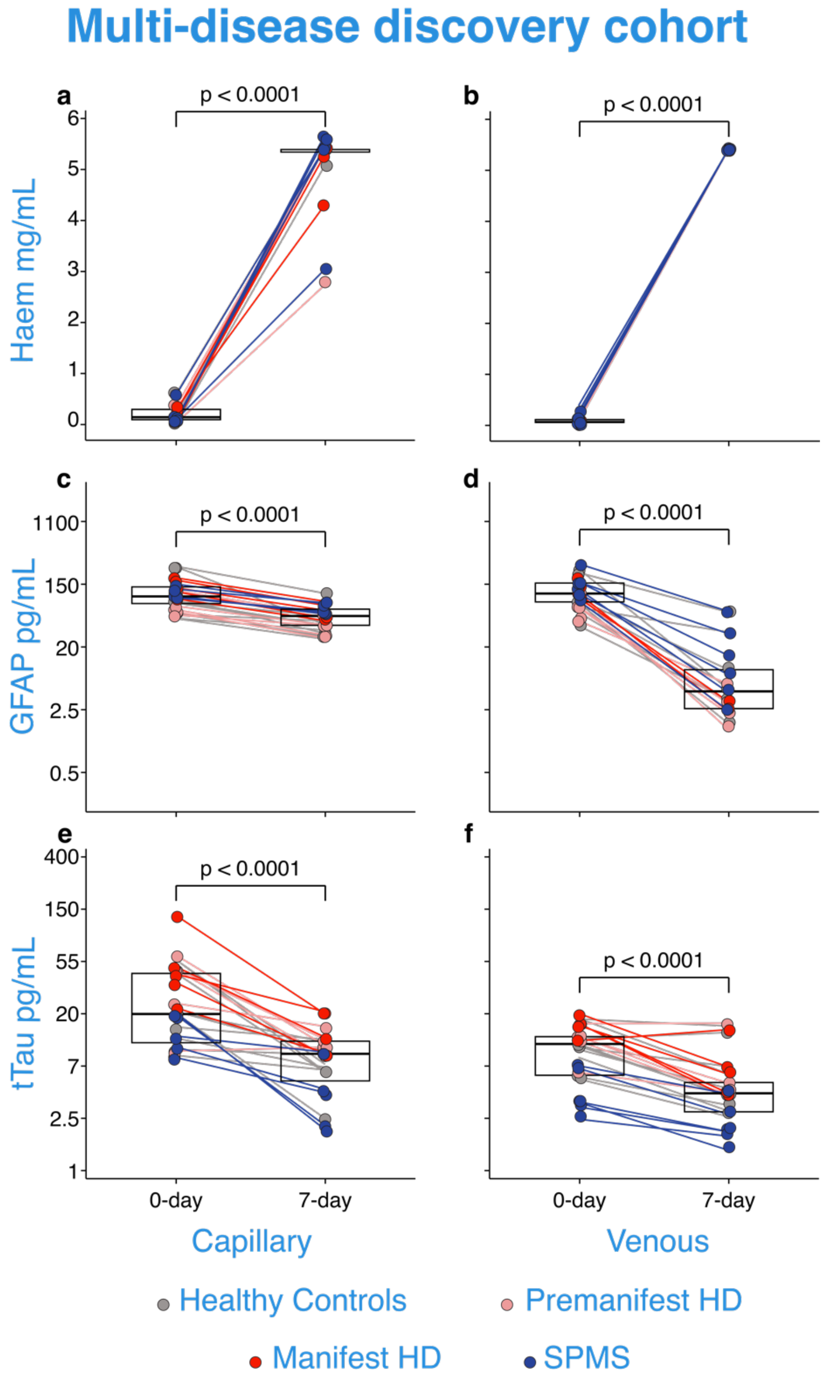

Fig. 12 *Concentrations of haemoglobin (a-b), GFAP (c-d), and tTau (e-f) from capillary and venous plasma in the multi-disease discovery cohort when processed on the day of collection and when processed seven days later from experiment B.1.b. p-values were generated from paired t-tests. Boxes represent the interquartile range with bold black horizontal line indicating the median. GFAP and tTau concentrations are natural log transformed. GFAP, glial fibrillary acidic protein; Haem, haemoglobin; HD, Huntington's disease; SPMS, secondary progressive multiple sclerosis; tTau, total tau*

<u>Fig. 13 – GFAP group comparisons for all baseline data in multi-disease discovery cohort</u>

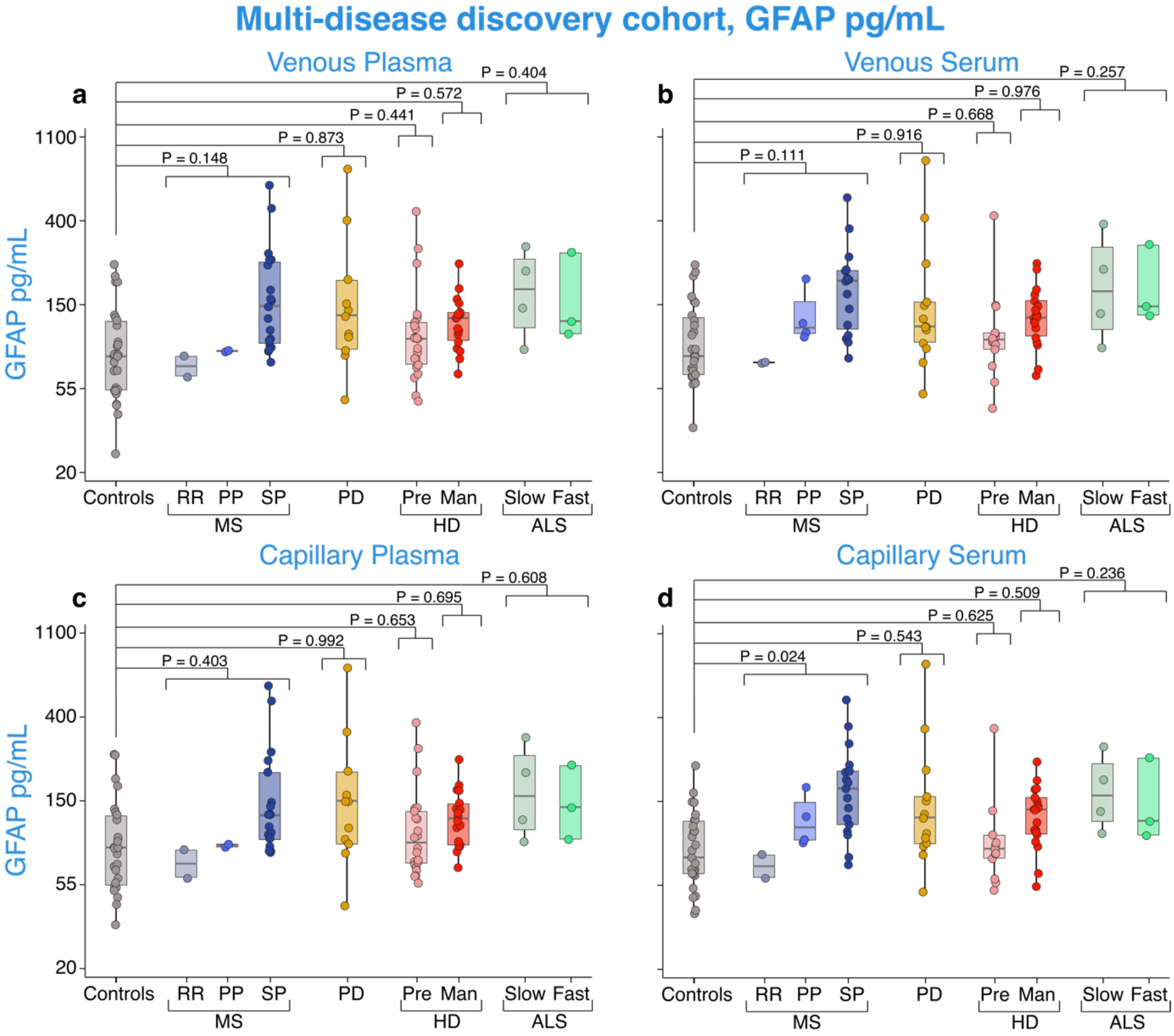

***Fig. 13** Disease group comparison of GFAP concentrations in the multi-disease discovery cohort from venous plasma (a), venous serum (b), capillary plasma (c), and capillary serum (d) from all baseline data for healthy controls, PPMS, RRMS, SPMS, PD, pre-HD, manifest-HD, ALS-slow and ALS-fast patients. P values were generated from multiple linear regressions. The Bonferroni threshold for this experiment was 0.0025 (20 comparisons) and all statistics which reached significance below this are highlighted in bold. GFAP concentrations are natural log transformed. ALS, amyotrophic lateral sclerosis; GFAP, glial fibrillary acidic protein; HD, Huntington's disease; Man, manifest; MS, multiple sclerosis; PD, Parkinson's disease; PP, primary progressive; Pre, premanifest; RR, relapsing-remitting; SP, secondary progressive*

Fig. 14 – A.0.0 results replicated in HD confirmatory cohort

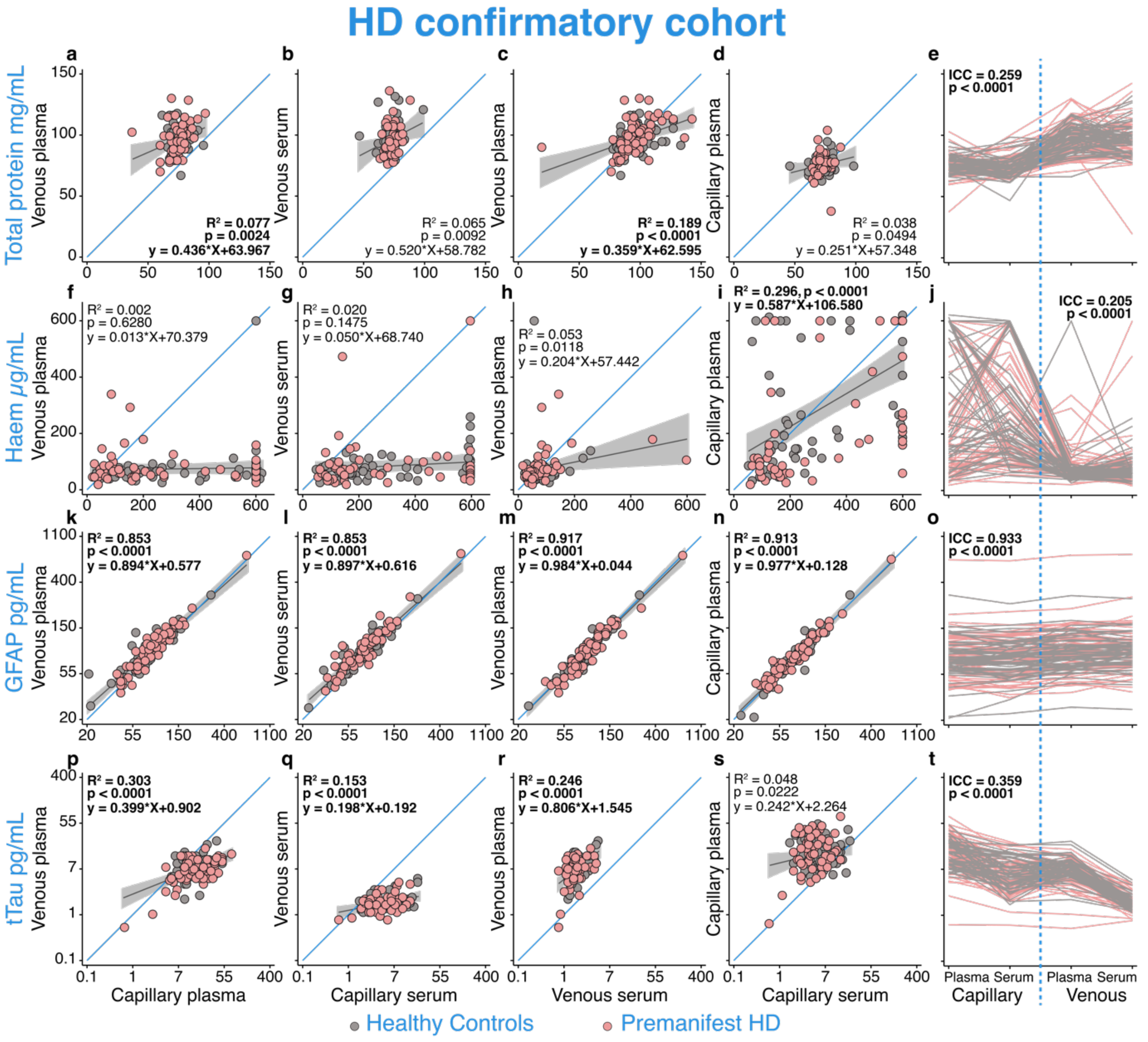

Fig. 14 *Analyte concentrations across different collection methods and sample types from the HD confirmatory cohort for total protein (a-e), haemoglobin (f-j), GFAP (k-o), and tTau (p-t) in healthy controls and pre-HD individuals. Blue lines represent y=x. Grey lines represent the linear regression fit of the data with 95% confidence interval shaded regions. R^2 and p-values were generated from regression models comparing the two collection types in each panel. The Bonferroni threshold for this experiment was 0.003125 (16 comparisons) and all statistics which reached significance below this are highlighted in bold. GFAP and tTau concentrations were natural log-transformed. GFAP, glial fibrillary acidic protein; Haem, haemoglobin; HD, Huntington's disease; tTau, total tau*

Fig. 15 – Capillary plasma NfL group comparisons in the HD confirmatory cohort and CAG-Age-NfL plot

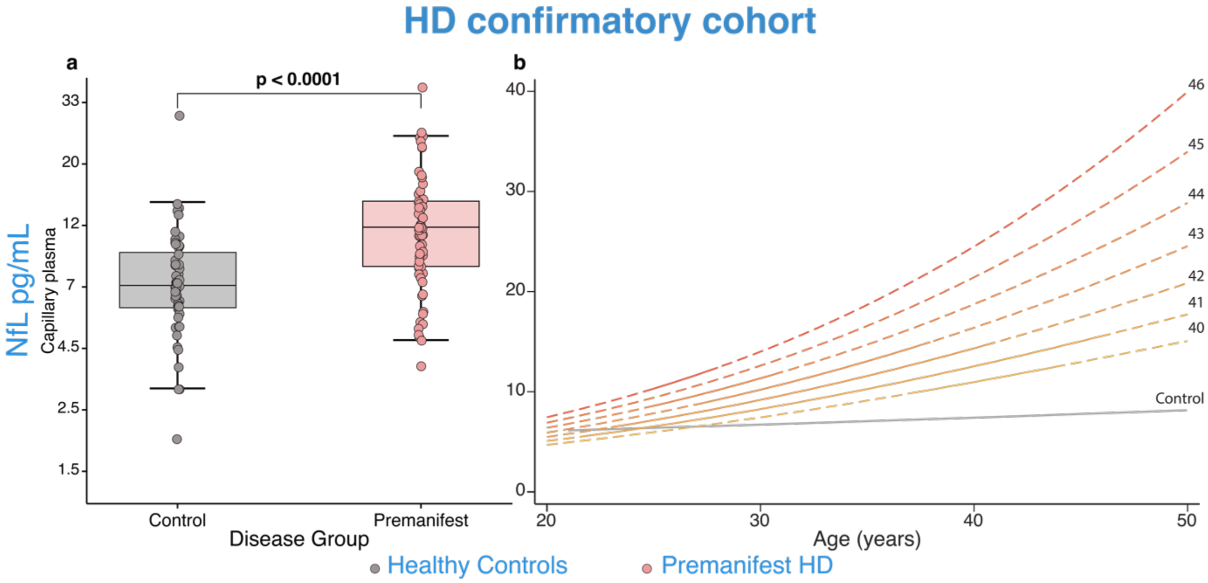

Fig. 15 *(a) Disease group differences between healthy controls and pre-HD in the HD confirmatory cohort from capillary plasma samples. p-values were generated from multiple linear regressions. Boxes show first and third quartiles, the central band shows the median, and the whiskers show data within 1.5 IQR of the median. The Bonferroni threshold for this experiment was 0.025 (2 comparisons) and statistics which reached significance below this are highlighted in bold. NfL values were natural log-transformed. (b) Modelling CAG-Age-NfL relationships to show associations between NfL, age, and CAG repeat count using capillary plasma samples. Solid lines were produced from our observations using a multiple linear regression model accounting for the main effects of age, CAG, the quadratic effect of age, and the interaction between age and CAG; dashed lines are predictions outside the range of our observations. Separate figures with individual data points for each individual CAG repeat count are provided in Supplementary Fig. 17. HD, Huntington's disease; NfL, neurofilament light*

Fig. 16 – Individual CAG plots for CAG-NfL-Age models from capillary serum samples

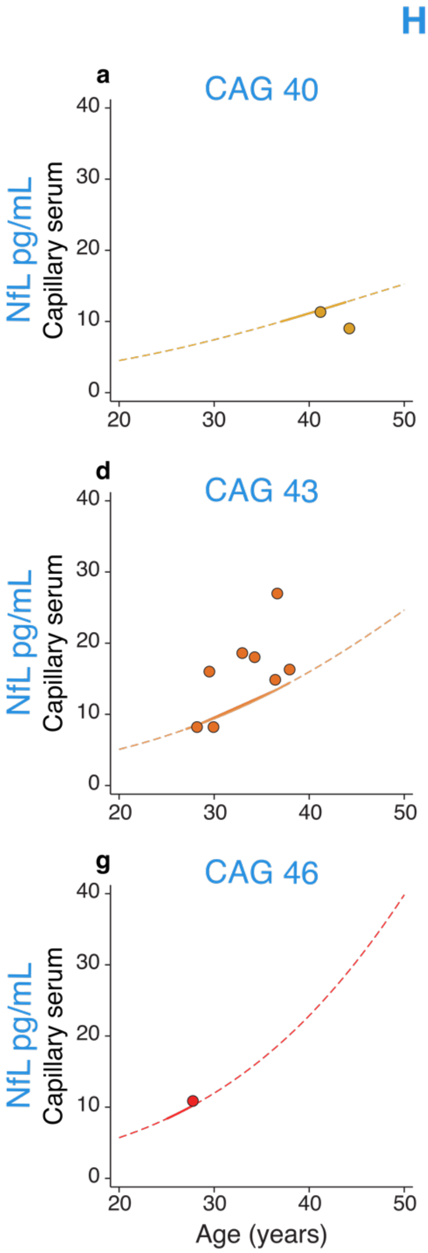

Fig. 16 *Individual CAG models of CAG-Age-NfL relationships in HD individuals from capillary serum samples in the HD confirmatory cohort to show associations between NfL, age, and CAG repeat count. Solid lines were produced from our observations using a multiple linear regression model accounting for the main effects of age, CAG, the quadratic effect of age, and the interaction between age and CAG; dashed lines are predictions outside the range of our observations. HD, Huntington's disease; NfL, neurofilament light*

Fig. 17 – Individual CAG plots for CAG-NfL-Age models from capillary plasma samples

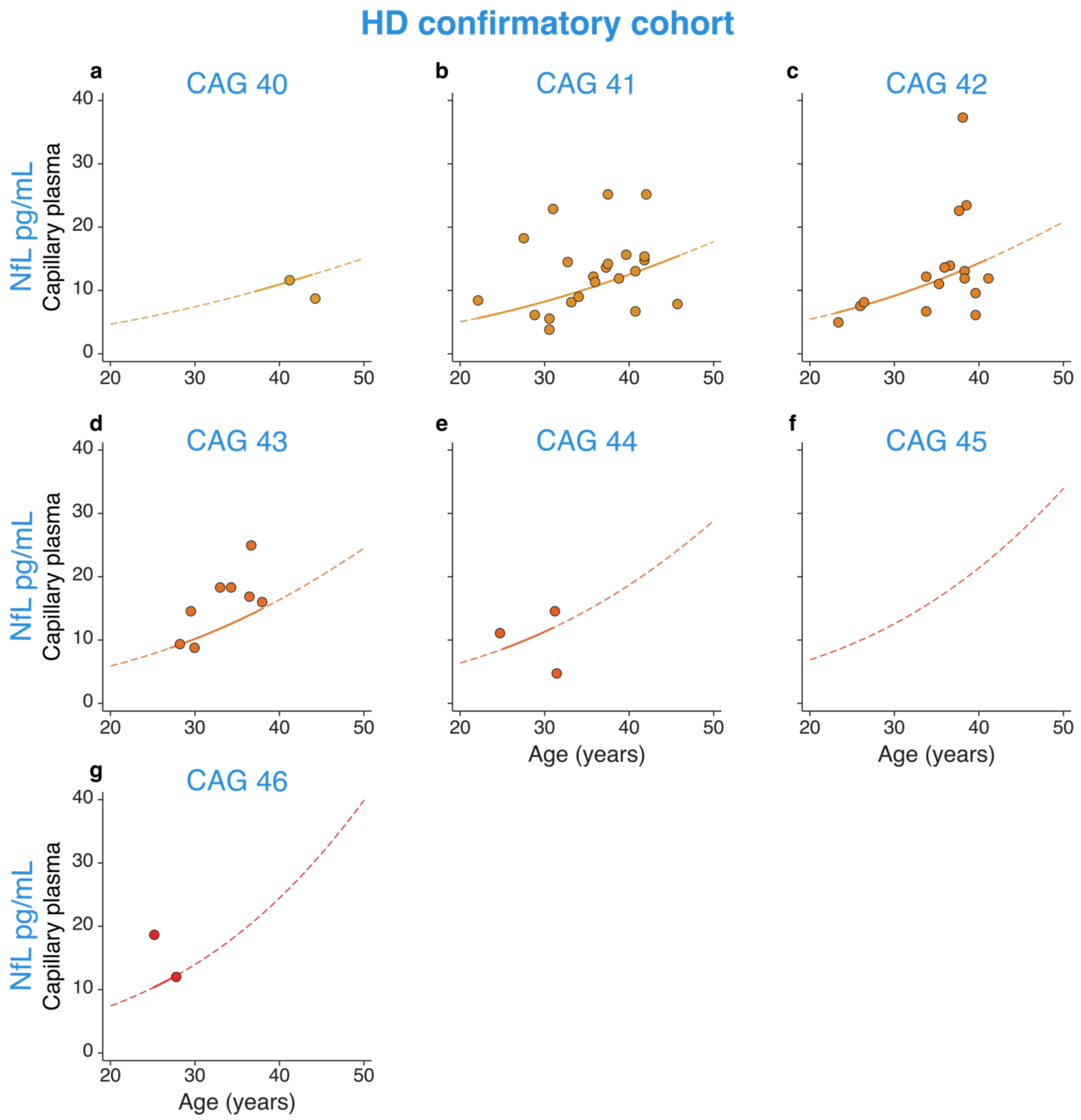

Fig. 17 *Individual CAG models of CAG-Age-NfL relationships in HD individuals from capillary plasma samples in the HD confirmatory cohort to show associations between NfL, age, and CAG repeat count. Solid lines were produced from our observations using a multiple linear regression model accounting for the main effects of age, CAG, the quadratic effect of age, and the interaction between age and CAG; dashed lines are predictions outside the range of our observations. HD, Huntington's disease; NfL, neurofilament light*

Table 1 – Technical variation

Table 1: Assessment of technical variation for each analyte with inter-assay % CV and average intra-assay % CV for each analyte measured.

Analyte	Inter-assay %CV	Intra-assay %CV
**NfL**	16.03	4.59
**GFAP**	28	7.32
**Tau**	12.56	4.45
**Haemoglobin**	68.63	5.45
**Total protein**	23.3	2.99

Table 2 – Group demographics for the multi-disease discovery cohort and confirmatory cohort

Table 2: Group demographics for all combined baseline data followed by group demographics for each objective in both the multi-disease discovery cohort and the confirmatory cohort.

Multi-disease discovery cohort							
**Total**	Controls	PD	Pre-HD	HD	ALS	MS	p-value
**n**	54	11	27	29	7	34	-
**Age (median, IQR)**	36.4 (29.6 – 58.2)	64.8 (53 – 71.8)	46 (39.4 – 52.3)	58.5 (51.5 – 61.5)	62.8 (49.7 – 76.2)	57.3 (52 – 60.3)	<0.0005
**Sex (%M)**	40	45	13	59	86	38	0.0017
**A.0.0**							
**n**	13	11	6	8	7	13	-
**Age (median, IQR)**	35.2 (26.9 - 45.8)	64.8 (53 – 71.8)	50.1 (36.9 – 59.6)	60.7 (50.4 – 62.2)	62.8 (49.7 – 76.2)	52 (48.3 – 56.8)	0.0015
**Sex (%M)**	38	45	17	50	86	38	0.2083
**B.1.a**							
**n**	11	-	6	5	-	5	-
**Age (median, IQR)**	31 (28.6 – 34.5)	-	49.2 (46.5 – 53.9)	51.5 (48 – 58.1)	-	59.6 (57,5 – 60.3)	0.0244
**Sex (%M)**	45	-	0	80	-	60	0.0162
**B.1.b**							
**n**	10	-	5	5	-	6	-
**Age (median, IQR)**	32.1 (30.8 – 61)	-	42.2 (40.3 – 52.3)	59.3 (52.5 – 60.3)	-	59.7 (59.3 – 60.4)	0.0936
**Sex (%M)**	40	-	20	20	-	33	0.8812
**B.2.a**							
**n**	10	-	5	5	-	5	-
**Age (median, IQR)**	31.2 (22.9 – 54.9)	-	39.4 (31.7 – 41.3)	58.5 (57.8 – 68.9)	-	59.1 (54.5 – 60.2)	0.0202
**Sex (%M)**	50	-	0	60	-	20	0.2346
**B.2.b**							
**n**	10	-	5	6	-	5	-
**Age (median, IQR)**	32.7 (27.5 – 48.5)	-	45.4 (44.7 – 54.3)	57.4 (46.9 – 61.5)	-	55.2 (55.1 – 66.5)	0.0136
**Sex (%M)**	20	-	20	83	-	40	0.0638
**HD confirmatory cohort**							
	Controls	PD	Pre-HD	HD	ALS	MS	p-value
**n**	57	-	64	-	-	-	-
**Age (median, IQR)**	33.7 (6.3)	-	33.9 (5.7)	-	-	-	0.8602
**Sex (%M)**	47	-	50	-	-	-	0.7725

ALS and MS disease subgroups were combined for groupwise comparisons. For the multi-disease discovery group, data are median (IQR) for age and % male for sex, P-values for age from the Kruskal-Wallis test and for sex from the chi-squared test. For the HD confirmatory cohort, data are mean (SD) for age and % male for sex, P-values for age are from an unpaired two-sample t-test and sex from a chi-squared test. ALS, amyotrophic lateral sclerosis; HD, Huntington's disease; MS, multiple sclerosis; PD, Parkinson's disease.

Tables 3 – 7: Summary statistics for each analyte in the multi-disease discovery cohort and HD confirmatory cohort

Table 3: Summary measures for NfL pg/mL from each objective in the multi-disease discovery cohort and for the HD confirmatory cohort.

NfL pg/mL<br>Multi-disease discovery cohort								
**A.0.0**		Controls	PD	Pre-HD	Manifest-HD	ALS	MS
Venous	Plasma	10.07 (5.76)	22.29 (10.41)	20.19 (9.39)	42.8 (13.5)	78.75 (64.19)	13.8 (6.86)
	Serum	9.79 (5.39)	21.82 (9.85)	18.9 (9.91)	42.67 (15.09)	91.19 (75.73)	14.02 (6.57)
Capillary	Plasma	12.69 (8.38)	24.09 (10.59)	20.29 (9.88)	48.84 (18.32)	80.86 (74.72)	15.13 (6.92)
	Serum	10.57 (5.93)	22.32 (9.56)	17.43 (9.64)	44.72 (16.14)	80.71 (71.89)	15.79 (7.53)
**B.1.a**							
Venous	Plasma day 0	7.36 (4.03)	-	24.09 (7.76)	52.48 (13.63)	-	17.07 (7.35)
	Plasma day 3	7.13 (3.64)	-	24.53 (8.42)	53.28 (14.75)	-	15.89 (7.39)
Capillary	Plasma day 0	10.38 (10.41)	-	24.9 (8.73)	53.28 (13.28)	-	17.77 (7.04)
	Plasma day 3	7.76 (4.14)	-	32.73 (9.49)	54.33 (15.81)	-	18.66 (8.42)
**B.1.b**							
Venous	Plasma day 0	9.93 (5.58)	-	31.91 (21.55)	54.25 (27.58)	-	17.59 (8.67)
	Plasma day 7	10.51 (6.38)	-	27.82 (20.91)	45.64 (22.42)	-	18.25 (11.32)
Capillary	Plasma day 0	10.97 (5.48)	-	31.79 (24.51)	53.71 (25.55)	-	12.29 (3.52)
	Plasma day 7	10.08 (5.23)	-	31.33 (24.77)	48.93 (25.33)	-	12.01 (3.12)
**B.2.a**							
Venous	Serum day 0	10.38 (5.21)	-	21.18 (10.04)	45.38 (13.75)	-	17.5 (4.76)
	Serum day 3	10.44 (5.7)	-	22.37 (11.59)	47.35 (13.93)	-	17.71 (4.31)
Capillary	Serum day 0	10.31 (4.79)	-	19.52 (8.73)	43.2 (11.85)	-	16.48 (4.72)
	Serum day 3	10.85 (4.88)	-	21.82 (9.84)	43.76 (11.61)	-	17.11 (5.04)
**B.2.b**							
Venous	Serum day 0	7.92 (3.74)	-	19.15 (9.26)	49.29 (27.22)	-	17.15 (5.76)
	Serum day 7	7.42 (3.37)	-	19.23 (8.83)	42.4 (23.76)	-	15.91 (4.62)
Capillary	Serum day 0	7.64 (5.43)	-	19.97 (9.2)	48.15 (26.06)	-	16.97 (4.63)
	Serum day 7	8.49 (7.64)	-	18.48 (8.99)	43.82 (24.17)	-	15.7 (3.62)
**HD confirmatory cohort**							
Venous	Plasma	7.2 (1.44)	-	11.89 (1.59)	-	-	-
	Serum	6.86 (1.46)	-	11.75 (1.63)	-	-	-
Capillary	Plasma	7.5 (1.52)	-	11.43 (1.6)	-	-	-
	Serum	6.97 (1.52)	-	10.8 (1.68)	-	-	-

Data are mean (SD). ALS, amyotrophic lateral sclerosis; HD, Huntington's disease; MS, multiple sclerosis; NfL, neurofilament light; PD, Parkinson's disease.

Table 4: Summary measures for GFAP pg/mL from each objective in the multi-disease discovery cohort and HD confirmatory cohort.

**GFAP pg/mL**							
**Multi-disease discovery cohort**							
**A.0.0**		Controls	PD	Pre-HD	Manifest-HD	ALS	MS
Venous	Plasma	97 (49.54)	199.3 (205.9)	153.5 (150.5)	129.6 (55.51)	178.9 (85.12)	160.6 (160.1)
	Serum	103.2 (53.79)	208.3 (230.8)	152.8 (139.7)	131.1 (53.77)	202.3 (108.4)	163.1 (132.9)
Capillary	Plasma	95.58 (47.14)	194.5 (193.5)	137.7 (121.2)	135.1 (55.36)	170.3 (82.89)	154 (143.8)
	Serum	90.66 (37.31)	194.4 (207.5)	140.6 (122.9)	131 (57.6)	168.3 (74.98)	154 (124.6)
**B.1.a**							
Venous	Plasma day 0	66.42 (34.42)	-	134.2 (78.21)	109.8 (23.54)	-	215.8 (152.1)
	Plasma day 3	27.72 (20.4)	-	51.87 (33.1)	66.27 (14.62)	-	71.25 (56.61)
Capillary	Plasma day 0	66.55 (34.96)	-	130.3 (71.27)	99.73 (21.39)	-	206.4 (167.1)
	Plasma day 3	54.21 (24.9)	-	79.13 (9.92)	85.82 (16.7)	-	142.5 (105.5)
**B.1.b**							
Venous	Plasma day 0	95.8 (70.56)	-	70.41 (25.18)	134.2 (32.76)	-	152.2 (65.98)
	Plasma day 7	12.29 (19.23)	-	2.77 (2.37)	1.27 (1.77)	-	20.56 (22.33)
Capillary	Plasma day 0	107.7 (80.68)	-	75.12 (25.88)	136.7 (35.46)	-	113.5 (21.68)
	Plasma day 7	44.27 (23.69)	-	37.94 (11.32)	63.15 (15.8)	-	66.59 (11.19)
**B.2.a**							
Venous	Serum day 0	114.2 (57.03)	-	94.55 (36.34)	124.2 (40.87)	-	195.9 (108)
	Serum day 3	93.15 (62.88)	-	88.82 (38.29)	119.6 (31.98)	-	195.5 (124.5)
Capillary	Serum day 0	109.9 (51.37)	-	89.57 (30.69)	119.1 (37.36)	-	189.7 (102.6)
	Serum day 3	106.3 (48.77)	-	89.23 (23.57)	118.3 (41.6)	-	186.8 (116.2)
**B.2.b**							
Venous	Serum day 0	82.71 (37.71)	-	92.35 (32.74)	148.5 (45.38)	-	192.9 (32.19)
	Serum day 7	33.11 (14.88)	-	57.8 (21.83)	67.03 (21.27)	-	97.64 (17.92)
Capillary	Serum day 0	69.61 (29.57)	-	77.71 (18.7)	135 (39.46)	-	184.1 (45.18)
	Serum day 7	48.96 (23.47)	-	54.94 (14.54)	82.83 (33.87)	-	104.2 (28.06)
**HD confirmatory cohort**							
Venous	Plasma	86.49 (1.48)	-	91.1 (1.63)	-	-	-
	Serum	87.88 (1.46)	-	94.16 (1.61)	-	-	-
Capillary	Plasma	92.76 (1.56)	-	81.04 (1.6)	-	-	-
	Serum	76.02 (1.51)	-	76.78 (1.66)	-	-	-

Data are mean (SD). ALS, amyotrophic lateral sclerosis; GFAP, glial fibrillary acidic protein; HD, Huntington's disease; MS, multiple sclerosis; PD, Parkinson's disease.

Table 5: Summary measures for tTau pg/mL from each objective in the multi-disease discovery cohort and HD confirmatory cohort.

tTau pg/mL							
**Multi-disease discovery cohort**							
**A.0.0**		Controls	PD	Pre-HD	Manifest-HD	ALS	MS
Venous	Plasma	8.26 (1.62)	7.94 (2.01)	12.47 (4.87)	11.48 (4.2)	1.84 (0.56)	5.05 (3.13)
	Serum	2.13 (1.38)	6.3 (0.72)	1.94 (0.51)	2.71 (1.51)	1.08 (0.1)	2.34 (1.94)
Capillary	Plasma	25.33 (16.24)	25.97 (13.67)	25.41 (9.33)	25.01 (15.19)	7.5 (5.74)	14.43 (6.98)
	Serum	6.49 (2.41)	15.3 (6.34)	4.35 (2.91)	5.08 (2.48)	2 (0.31)	8.07 (6)
**B.1.a**							
Venous	Plasma day 0	7.64 (3.39)	-	10.39 (2.61)	6.7 (2.28)	-	5.33 (4.03)
	Plasma day 3	3.89 (2.09)	-	4.6 (2.25)	3.22 (1.06)	-	2.34 (0.76)
Capillary	Plasma day 0	20.11 (15.96)	-	25.96 (13.86)	18.36 (10.93)	-	22.24 (27.22)
	Plasma day 3	13.14 (10.53)	-	15.02 (9.59)	5.01 (1.93)	-	8.78 (4.4)
**B.1.b**							
Venous	Plasma day 0	11.23 (4.17)	-	12.22 (3.53)	16.17 (2.82)	-	4.8 (2.04)
	Plasma day 7	6.69 (4.55)	-	7.12 (5.31)	7.43 (4.31)	-	2.6 (1.04)
Capillary	Plasma day 0	25.74 (17.87)	-	37.12 (20.08)	55.31 (42.4)	-	13.94 (5.01)
	Plasma day 7	7.83 (2.76)	-	11.41 (2.55)	14.14 (5.57)	-	4.52 (2.95)
**B.2.a**							
Venous	Serum day 0	2.2 (0.99)	-	2.53 (0.81)	2.37 (1.49)	-	2 (1.26)
	Serum day 3	3.4 (1.27)	-	3.36 (1.01)	2.86 (1.52)	-	2.34 (1.21)
Capillary	Serum day 0	8.86 (4.87)	-	9.27 (4.19)	7.83 (4.25)	-	7.59 (6.21)
	Serum day 3	11.5 (8.92)	-	15.63 (10.58)	4.16 (1.82)	-	7.56 (4.24)
**B.2.b**							
Venous	Serum day 0	2.1 (0.85)	-	3.12 (1.7)	2.52 (1.71)	-	1.52 (0.43)
	Serum day 7	4.02 (1.1)	-	5.03 (2.41)	2.89 (1.37)	-	2.02 (0.58)
Capillary	Serum day 0	10.06 (8.79)	-	7.73 (4.85)	6.02 (1.97)	-	8.21 (5.51)
	Serum day 7	5.99 (2.57)	-	8.14 (5.59)	8.07 (4.22)	-	5.9 (1.54)
**HD confirmatory cohort**							
Venous	Plasma	7.61 (1.59)	-	6.82 (1.8)	-	-	-
	Serum	1.76 (1.35)	-	1.64 (1.42)	-	-	-
Capillary	Plasma	13.92 (1.78)	-	15.78 (2.31)	-	-	-
	Serum	6.08 (1.9)	-	4.22 (1.86)	-	-	-

Data are mean (SD). ALS, amyotrophic lateral sclerosis; HD, Huntington's disease; MS, multiple sclerosis; PD, Parkinson's disease; tTau, total tau.

Table 6: Summary measures for total protein mg/mL from each objective in the multi-disease discovery cohort and HD confirmatory cohort.

**Total protein mg/mL**							
**Multi-disease discovery cohort**							
**A.0.0**		Controls	PD	Pre-HD	Manifest-HD	ALS	MS
Venous	Plasma	76.79 (6.267)	80.55 (6.88)	78.64 (12.89)	72.63 (8.91)	81.27 (5.9)	93.67 (15.96)
	Serum	76.2 (5.41)	77.82 (3.35)	72.39 (5.93)	70.16 (6.35)	78.96 (7.27)	90.01 (8.812)
Capillary	Plasma	69.51 (5.28)	74.02 (6.19)	67.5 (4.12)	67.67 (5.54)	73.71 (6.51)	76.98 (18.77)
	Serum	69.33 (7.56)	71.63 (3.97)	65.75 (3.47)	68.11 (5.68)	66.53 (1.99)	67.15 (4.96)
**B.1.a**							
Venous	Plasma day 0	83.11 (10.63)	-	74.33 (19.56)	79.12 (14.38)	-	83.42 (10.51)
	Plasma day 3	89.31 (12.51)	-	87.91 (5.99)	78.29 (4.46)	-	88.4 (10.06)
Capillary	Plasma day 0	68.24 (5.04)	-	62.74 (12.22)	62.83 (4.96)	-	72.11 (6.68)
	Plasma day 3	76.75 (8.77)	-	78.34 (7.42)	70.23 (5.53)	-	75.52 (3.89)
**B.1.b**							
Venous	Plasma day 0	78.41 (19.14)	-	86.65 (6.67)	81.73 (9.5)	-	81.38 (5.26)
	Plasma day 7	80.09 (8.08)	-	82.59 (17.24)	77.58 (11.04)	-	85.07 (7.2)
Capillary	Plasma day 0	67.84 (4.1)	-	68.37 (4.06)	64.57 (8.99)	-	71.78 (5.75)
	Plasma day 7	65.14 (5.63)	-	67.79 (6.98)	64.94 (7.14)	-	70.14 (3.83)
**B.2.a**							
Venous	Serum day 0	93.54 (15.98)	-	78.7 (10.38)	89.95 (9.15)	-	83.4(8.62)
	Serum day 3	92.41 (20.07)	-	106.2 (16.9)	88.66 (8.9)	-	88.46 (12.99)
Capillary	Serum day 0	63.25 (8.99)	-	62.16 (6.23)	72.61 (1.56)	-	69.91 (5.42)
	Serum day 3	66.44 (5.76)	-	65.44 (7.08)	62.57 (6.05)	-	70.54 (2.68)
**B.2.b**							
Venous	Serum day 0	78.44 (10.15)	-	89.25 (14.24)	77.55 (9.26)	-	91.07 (10.73)
	Serum day 7	72.08 (10.4)	-	82.52 (12.9)	79.09 (10.42)	-	92.96 (13.95)
Capillary	Serum day 0	63.14 (8.6)	-	62.78 (4.35)	61.13 (7.02)	-	69.48 (4.08)
	Serum day 7	58.38 (12.38)	-	56.22 (3.61)	57.42 (3.92)	-	68.2 (4.44)
**HD confirmatory cohort**							
Venous	Plasma	96.6 (10.39)	-	97.48 (13.77)	-	-	-
	Serum	97.32 (12.2)	-	94.65 (16.73)	-	-	-
Capillary	Plasma	75.65 (6.45)	-	74.93 (9.26)	-	-	-
	Serum	72.49 (7.4)	-	72.75 (4.74)	-	-	-

Data are mean (SD). ALS, amyotrophic lateral sclerosis; HD, Huntington's disease; MS, multiple sclerosis; PD, Parkinson's disease.

Table 7: Summary measures for haemoglobin ng/mL from each objective in the multi-disease discovery cohort and HD confirmatory cohort.

Haemoglobin ng/mL<br>Multi-disease discovery cohort								
**A.0.0**		Controls	PD	Pre-HD	Manifest-HD	ALS	MS
Venous	Plasma	56.35 (16.88)	64.88 (71.54)	91.9 (70.22)	72.26 (31.82)	66.43 (44.16)	143.5 (169.8)
	Serum	61.06 (44.82)	157.3 (163.7)	112.9 (182.5)	55.24 (18.38)	102.5 (81.91)	193.5 (206)
Capillary	Plasma	296.4 (243.6)	505.1 (180.1)	318.3 (231.2)	211.7 (195.6)	284.5 (233.4)	235 (204.8)
	Serum	304.9 (222.4)	583.7 (49.78)	306.8 (200.1)	198.7 (116.9)	439.6 (228.1)	380.2 (200.5)
**B.1.a**							
Venous	Plasma day 0	64.94 (29.2)	-	73.73 (51.03)	45.78 (21.24)	-	158.3 (232.8)
	Plasma day 3	296.4 (103)	-	243.2 (114.5)	394.1 (141.5)	-	403.2 (162.2)
Capillary	Plasma day 0	110.9 (75.53)	-	244.9 (208.6)	169.1 (225.8)	-	193 (131.6)
	Plasma day 3	283.2 (184.8)	-	365 (215.1)	291.5 (223.1)	-	332.2 (163.8
**B.1.b**							
Venous	Plasma day 0	121.6 (173.6)	-	62.88 (31.99)	80.16 (30.33)	-	111 (93.68)
	Plasma day 7	5400 (0)	-	5400 (0)	5400 (0)	-	5400 (0)
Capillary	Plasma day 0	222.3 (215.4)	-	272.2 (214.8)	175.7 (122.2)	-	206.2 (222.3)
	Plasma day 7	4120 (1684)	-	4873 (1178)	5153 (479.9)	-	5017 (1098)
**B.2.a**							
Venous	Serum day 0	89.25 (60.34)	-	46.32 (16.4)	98.34 (131.1)	-	224.3 (212.4)
	Serum day 3	399.5 (177.7)	-	110 (49.67)	349.1 (251.7)	-	489.7 (187)
Capillary	Serum day 0	233.7 (146.6)	-	223.9 (223.2)	324.5 (174.4)	-	244.1 (118.8)
	Serum day 3	310.5 (240)	-	162.8 (138.4)	440.2 (219)	-	235.8 (215.8)
**B.2.b**							
Venous	Serum day 0	53.43 (17.45)	-	260 (261.1)	97.95 (81.28)	-	66 (36.59)
	Serum day 7	5300 (334.1)	-	5400 (0)	4827 (1455)	-	5400 (0)
Capillary	Serum day 0	328.6 (216)	-	355.4 (233.8)	489.9 (145)	-	304.7 (233.4)
	Serum day 7	4420 (1575)	-	4690 (910.2)	5058 (838.8)	-	4963 (414.2)
**HD confirmatory cohort**							
Venous	Plasma	68.89 (74.32)	-	79.03 (53.18)	-	-	-
	Serum	78.37 (44.38)	-	84.84 (89.45)	-	-	-
Capillary	Plasma	335 (221.1)	-	279.7 (230.6)	-	-	-
	Serum	317.8 (201.4)	-	319.2 (217.6)	-	-	-

Data are mean (SD). ALS, amyotrophic lateral sclerosis; HD, Huntington's disease; MS, multiple sclerosis; PD, Parkinson's disease.

Table 8 – Mean differences for multi-disease discovery cohort for NfL

Table 8: Mean differences for all baseline NfL data in the multi-disease discovery cohort.

NfL pg/mL		PD – controls		Pre-HD – controls		Manifest-HD – controls		ALS – controls		MS – controls	
		(95% CI)	*p*	(95% CI)	*p*	(95% CI)	*p*	(95% CI)	*p*	(95% CI)	*p*
Venous	Plasma	11.26 (0.11,0.83)	0.010	**13.63 (0.59, 1.11)**	**< 0.0001**	**36.63 (1.16, 1.73)**	**< 0.0001**	**52.91 (1.2, 2.02)**	**< 0.0001**	5.11 (-0.23, 0.52)	0.072
	Serum	8.64 (-0.01, 0.66)	0.051	**8.17 (0.33, 0.89)**	**< 0.0001**	**33 (1.01, 1.57)**	**< 0.0001**	**59.9 (1.31, 2.1)**	**< 0.0001**	5.35 (-0.02, 0.51)	0.067
Capillary	Plasma	11.65 (0.06, 0.88)	0.024	**7.05 (0.41, 1.01)**	**< 0.0001**	**37.47 (1.05, 1.71)**	**< 0.0001**	**49.8 (1.03, 1.97)**	**< 0.0001**	3.48 (-0.18, 0.44)	0.396
	Serum	**9.13 (0.06, 0.76)**	**0.0023**	**7.16 (0.25, 0.88)**	**0.001**	**32.82 (1.02, 1.62)**	**< 0.0001**	**51.51 (1.2, 2.05)**	**< 0.0001**	5.81 (0.37, 0.6)	0.027

ALS and MS disease subgroups were combined for groupwise comparisons. Data are mean difference (95% CI), p-value. P-values were generated from multiple linear regressions. The Bonferroni threshold for this experiment was 0.0025 (20 comparisons) and all statistics which reached significance below this are highlighted in bold. ALS, amyotrophic lateral sclerosis; CI, confidence interval; HD, Huntington's disease; MS, multiple sclerosis; NfL, neurofilament light; PD, Parkinson's disease.

Table 9 – Mean differences for multi-disease discovery cohort for GFAP

Table 9: Mean differences for all baseline GFAP data in the multi-disease discovery cohort.

GFAP pg/mL		PD – controls		Pre-HD – controls		Manifest-HD – controls		ALS – controls		MS – controls	
		(95% CI)	*p*	(95% CI)	*p*	(95% CI)	(95% CI)	*p*	(95% CI)	*p*	(95% CI)
Venous	Plasma	103.45 (-0.34, 0.4)	0.873	28.25 (-0.16, 0.37)	0.441	29.55 (-0.21, 0.38)	0.572	83.05 (-0.24, 0.6)	0.404	74.55 (-0.07, 0.48)	0.148
	Serum	104.6 (-0.35, 0.12)	0.916	16.4 (-0.23, 0.36)	0.668	31.1 (-0.29, 028)	0.976	98.6 (-0.17, 0.64)	0.257	73.6 (-0.05, 0.49)	0.111
Capillary	Plasma	95.7 (-0.35, 0.35)	0.992	19.9 (-0.2, 0.32)	0.653	26.9 (-0.23, 0.34)	0.695	71.5 (-0.3, 0.51)	0.608	57.8 (-0.15, 0.38)	0.403
	Serum	103.21 (-0.23, 0.44)	0.543	13.21 (-0.23, 0.37)	0.625	37.91 (-0.19, 0.38)	0.509	77.11 (-0.16, 0.64)	0.236	77.11 (0.04, 0.58)	0.024

ALS and MS disease subgroups were combined for groupwise comparisons. Data are mean difference (95% CI), p-value. P-values were generated from multiple linear regressions. The Bonferroni threshold for this experiment was 0.0025 (20 comparisons) and all statistics which reached significance below this are highlighted in bold. CI, confidence interval; ALS, amyotrophic lateral sclerosis; GFAP, glial fibrillary acidic protein; HD, Huntington's disease; MS, multiple sclerosis; PD, Parkinson's disease.

Table 10: Mean differences for HD confirmatory cohort for NfL

Table 10: Mean differences for NfL data in the HD confirmatory cohort.

NfL pg/mL		Pre-HD – controls	
		(95% CI)	*p*
Venous	Plasma	**1.71 (1.46, 1.98)**	**< 0.0001**
	Serum	**1.65 (1.43, 1.9)**	**< 0.0001**
Capillary	Plasma	**1.52 (1.29, 1.77)**	**< 0.0001**
	Serum	**1.55 (1.3, 1.84)**	**< 0.0001**

Data are mean difference (95% CI), p-value. P-values were generated from multiple linear regressions. The Bonferroni threshold for this experiment was 0.0125 (4 comparisons) and all statistics which reached significance below this are highlighted in bold. CI, confidence interval; HD, Huntington's disease.

Table 11 – 12: Relationships between capillary serum NfL, age and CAG repeat in the HD confirmatory cohort

Table 11: Relationship between capillary serum NfL (log) age in control participants only from the HD confirmatory cohort.

Confirmatory cohort					
	Estimate	Standard error	95% CI	t-value	p-value
**Age**	0.01	0.0042	0.002 to 0.019	2.43	0.017
**Intercept**	1.57	0.165	1.238 to 1.897	9.47	< 0.0001

Model fit specifically to healthy control data in the HD confirmatory cohort data, accounting for the main effects of age. Statistics are from logged NfL values. CI, confidence interval.

Table 12: Relationship between capillary serum NfL (log), age, and CAG repeat count in participants with HD from the HD confirmatory cohort.

Confirmatory cohort					
	Estimate	Standard error	95% CI	t-value	p-value
**Intercept**	1.904	4.157	-6.33 to 10.14	0.46	0.648
**Age**	-0.089	0.097	-0.281 to 0.104	-0.91	0.364
**CAG**	-0.042	0.095	-0.229 to 0.146	-0.44	0.661
**Age x CAG**	0.004	0.0021	-0.0001 to 0.008	1.91	0.059
**Age^2**	-0.0005	0.00026	-0.001 to 0.0001	-1.75	0.083

Model fit specifically to pre-HD data in the HD confirmatory cohort data, accounting for the main effects of age, CAG, the quadratic effect of age, and the interaction between age and CAG. Statistics are from logged NfL values. CI, confidence interval.

Table 13 – 14: Relationships between capillary plasma NfL, age and CAG repeat in the HD confirmatory cohort

Table 13: Relationship between capillary plasma NfL (log) age in control participants only from the HD confirmatory cohort.

Confirmatory cohort					
	Estimate	Standard error	95% CI	t-value	p-value
**Intercept**	1.63	0.166	1.308 to 1.967	9.89	< 0.0001
**Age**	0.01	0.0042	0.001 to 1.308	2.32	0.023

Model fit specifically to healthy control data in the HD confirmatory cohort data, accounting for the main effects of age. Statistics are from logged NfL values. CI, confidence interval.

Table 14: Relationship between capillary plasma NfL (log), age, and CAG repeat count in participants with HD from the HD confirmatory cohort.

Confirmatory cohort					
	Estimate	Standard error	95% CI	t-value	p-value
**Intercept**	-0.372	3.804	-7.91 to 7.16	-0.10	0.922
**Age**	-0.051	0.091	-0.23 to 0.129	-0.56	0.578
**CAG**	0.198	0.086	-0.151 to 0.190	0.23	0.819
**Age x CAG**	0.003	0.002	-0.001 to 0.007	1.47	0.146
**Age^2**	-0.0004	0.0002	-0.0008 to 0.0001	-1.41	0.161

Model fit specifically to pre-HD data in the HD confirmatory cohort data, accounting for the main effects of age, CAG, the quadratic effect of age, and the interaction between age and CAG. Statistics are from logged NfL values. CI, confidence interval.
